# Computational analysis of transcriptome data and mapping of functional networks in Parkinson’s disease

**DOI:** 10.3389/fbinf.2025.1690229

**Published:** 2025-11-19

**Authors:** Konstantinos Perperidis, Themis P. Exarchos, Aristidis G. Vrahatis, Panagiotis Vlamos, Marios G. Krokidis

**Affiliations:** 1 Bioinformatics and Human Electrophysiology Laboratory, Department of Informatics, Ionian University, Corfu, Greece; 2 Institute of Digital Biomedicine, Ionian University Research and Innovation Center, Corfu, Greece

**Keywords:** Parkinson’s disease, machine learning, transcriptomics, functional networks, enrichment analysis, classification accuracy

## Abstract

Parkinson’s disease (PD) is the most common neurodegenerative movement disorder. The pathophysiology is defined by a loss of dopaminergic neurons in the substantia nigra pars compacta, however recent studies suggest that the peripheral immune system may participate in PD development. Herein, we analyzed molecular insights examining RNA-seq data obtained from the peripheral blood of both Parkinson’s disease patients and healthy control. Although all age and gender groups were analyzed, emphasis is given on individuals aged 50–70, the most prevalent group for Parkinson’s diagnosis. The computational workflow comprises both bioinformatics analyses and machine learning processes and the yield of the pipeline includes transcripts ranked by their level of significance, which could serve as reliable genetic signatures. Classification outcomes are also examined with a focus on the significance of selected features, ultimately facilitating the development of gene networks implicated in the disease. The thorough functional analysis of the most prominent genes, regarding their biological relevance to PD, indicates that the proposed framework has strong potential for identifying blood-based biomarkers of the disease. Moreover, this approach facilitates the application of machine learning techniques to RNA-seq data from complex disorders, enabling deeper insights into critical biological processes at the molecular level.

## 1 Introduction

Parkinson’s disease (PD), a neurological movement disorder most prominently characterized by tremors, was first formally described in 1817 by the British physician James Parkinson (Goetz, 2011; Balestrino and Schapira, 2020). Advancing age is recognized as the most significant risk factor, although genetic mutations have also been implicated. Furthermore, exposure to environmental toxins is considered a potential epigenetic contributor to the disease’s pathogenesis (Tsalenchuk et al., 2023). The hallmark motor symptoms include tremors, muscular rigidity, bradykinesia or akinesia, and postural instability. Diagnosis is primarily based on clinical evaluation, although additional tests may be utilized to aid in differential diagnosis (Shin et al., 2022). From an epidemiological perspective, PD is known to affect 1 to 2 individuals per 1,000 at any time, with a rising frequency proportionally to age and a prevalence of 1% for ages above 60 years. The prevalence for genetically linked cases of Parkinson’s disease is as low as 5%–15% of the total cases. Apart from progressed age, which may be considered a consensus risk factor across several studies, gender statistically plays a role in PD, affecting men more than women (Baldereschi et al., 2000; Van Den Eeden et al., 2003). Genetic predisposition is also considered a significant risk factor, as familial cases of Parkinson’s disease have been reported, suggesting a possible autosomal-dominant pattern of inheritance (Tanner and Goldman, 2005).

Several genes were identified to be crucially involved in the manifestation of PD phenotype. Among the most commonly known are SNCA, LRRK2, PRKN, PINK1, VPS35 and GBA1. The SNCA gene accounts for the translation of the protein alpha-synuclein, which, among other processes, is also involved in the release of neurotransmitters (Jankovic and Tan, 2020; Trevisan et al., 2024). Pathogenic mutations are uncommon but clearly cause hereditary and early-onset forms of the disease. Such mutations translate to a misfolded form of the protein which burdens its proper degradation and the accumulation within cells. The LRRK2 gene codes for a kinase which functions as an arbiter of neuronal processes. Despite its many variants, there are only a few known to be pathological. Pathologic variants are inherited in an autosomal dominant pattern with chances of disease manifestation of 30% at 50% and 70% at 80 years of age (Healy et al., 2008). The mutation is thought to play a role in mediating neuroinflammation, and studies have also explored potential interactions between LRRK2 and SNCA (Bieri et al., 2019). PRKN encodes the protein Parkin, an ingredient of a ubiquitin complex (Trevisan et al., 2024). Together with other proteins, like PINK1, it promotes the deconstruction of defected mitochondria (Yoshino et al., 2022)and is regarded as the most common autosomal recessive gene to account for up to 40% to disease onset at ages as young as 40 years (Wasner et al., 2022). Mutations cause Parkin protein misfolding, impairing its function and resulting in increased ubiquitination and compromised proteasome degradation.

Neurological specialists typically diagnose PD based on clinical symptoms; however, a definitive diagnosis distinguishing it from other neurodegenerative parkinsonian disorders requires post-mortem confirmation of Lewy bodies in the substantia nigra pars compacta (Miller and O’Callaghan, 2015). Only a limited number of established biomarkers are currently available to support the diagnosis of the disease. Cerebrospinal fluid is considered reliable due to the proximity to the central nervous system. Imaging approached such as PET, SPECT and MRI can provide valuable information not only about the structural composition of the brain but also its functional state.

The present study presents an analysis sequence that was employed on PPMI’s project 133 RNA Sequencing data of whole blood samples. From the wide range of groups available in the dataset, only the Parkinson’s disease (PD) and control cohorts were selected for analysis. The analysis sequence is structured as a pipeline incorporating a variety of computational techniques, including principal component analysis for exploratory data analysis and stratified differential gene expression, with each stratum representing distinct gender and age groups based on study participants. Additionally, sets of differentially expressed genes are utilized as features for selecting widely adopted machine learning algorithms. Moreover, an overview of the classification outcomes with respect to feature importance is provided, and ultimately, the development of gene networks hypothesized to influence Parkinson’s disease is addressed. This includes a gene set enrichment analysis (GSEA) conducted to assess the biological relevance of the findings. While analyses were conducted across all strata, this study concentrates on the most represented subgroup—males and females aged 50 to 70 years—as this demographic corresponds to the highest incidence of PD.

## 2 Methodology

### 2.1 Data

RNA sequencing data were used, originating from the Parkinson’s Progression Markers Initiative (PPMI) project 133 IR3 with the latest version of 4 February 2021. Transcriptome sequencing was conducted by the PPMI based on whole-blood samples, collected from Parkinson’s disease patients and healthy controls. The set of samples and the CSV metadata file were derived after registration on the PPMI’s study data dissemination provided by the Imaging and Data Archive, IDA, University of Southern California. The downloaded archive contains individual sample files as feature counts (Liao et al., 2014) and with TPM normalization (Zhao et al., 2021). A download in the FASTQ format is not possible online; instead, a hard drive can be requested to be shipped by the IDA from the USA after submission of a special request to the IDA. The size of the FASTQ file is about 184 Tera Bytes according to the project’s manual. Statistical analyses and machine learning modeling were implemented in Python and R. All scripts and relevant code are provided in Supplementary Table S5.

### 2.2 Data preparation and consolidation

The analysis conducted and presented in the present paper used feature counts as the only format available that can be considered close to raw counts, whereas TPM is not suitable for cross-sample analysis but rather within-sample. The downloaded archive contains individual files per sample. Based on the feature count set of files and the metadata CSV, a consolidated form of all the data available was created with an AnnData (Virshup et al., 2024) object. Since the metadata also includes the results of a quality check, annotating samples with either 'failed' or 'passed' columns, only the 'passed' ones were retained for downstream analysis. Since the samples are annotated by the participants’ gender and age as well (information that makes biologically sense), this information was used to stratify samples for downstream analysis, where each stratum is dedicated to a gender and the age groups of participants from PD’s and control cohorts. Age groups were set for 30–50, 50–70, 70–80 and >80 years of age. Supplementary Figure S25 presents the demographic composition (sex and age) of the dataset. Sample quality control was based exclusively on the PPMI ‘passed’ quality flag, with no additional exclusions required.

### 2.3 Data analysis

Principal component analysis was used in exploratory data analysis to check for technical bias that could affect downstream results. Several aspects that could induce perturbations were analyzed, with a focus on whether the variance of expressions is influenced by the fact that samples were gathered in two distinct phases and over several visits.

The dataset was split into several strata, with each stratum containing samples for a particular gender and age group, and differential expression analysis was conducted via the R program DESeq2 (Love et al., 2016) for all genders’ age groups. The visits were set as a covariate to the analysis. The thresholds were set at |log2FoldChange| >0.5 and padj <0.05. The fold change was set at 0.5 to increase the amount of potentially differentially expressed genes. The findings were validated by comparing them with the search feature of the Gene4PD website (Li et al., 2021) to evaluate their established biological relevance related to Parkinson’s disease. The results of statistically significant differentially expressed genes were exported into CSV files for further downstream analysis.

The statistically significant genes per stratum were used as features to train and assess machine learning models. In particular, the algorithms Logistic Regression, Support Vector Machine, Random Forest, and XGBoost were used. Each model was constructed by using 80% of the total samples of the respective stratum. The class imbalance ratio for each stratum is about 2:1, in favor of the case class; thus, the data can be considered as moderately imbalanced. Class weights were set to balanced in the classification models to address training imbalance. Due to the moderate degree of imbalance and to avoid overoptimistic predictions, no further actions were performed about that. Since samples were taken from the same individuals, the test and training subsets were divided by using group shuffle split, as provided by the Python library scikit (Buitinck et al., 2013). Hyperparameters were set for each model while the best set of parameters was decided via ten-fold cross-validation. Ten-fold cross-validation was also used to compare all algorithms across all strata. Predictions were run on the designated 20% part of the split-up dataset and the respective results exported for assessment. For all machine learning actions, the metrics ROC-AUC and PR-AUC were gathered, as well as sensitivity and recall scores, particularly to assess and compare prediction performance for all trained and tested models. The extraction and comparison of feature importances per ML model was achieved by employing SHAP analysis and plotting the respective beeswarm plots.

Functional networks were identified based on differentially expressed genes per stratum via the software package Cytoscape (Shannon et al., 2003). Furthermore, functional and publication enrichment was performed by using the STRING database (Szklarczyk et al., 2023) which API is used by respective interfaces within the Cytoscape software. Additionally, to the enrichment results from STRING, gene ontology databases as well as phenotype and transcription factor resources were consulted by using the GSEApy (Fang et al., 2023) library in Python. The following list provides an overview of the sources (gene sets) used to retrieve enrichment information from.

## 3 Results and discussion

### 3.1 Exploratory data analysis

**FIGURE 1:**
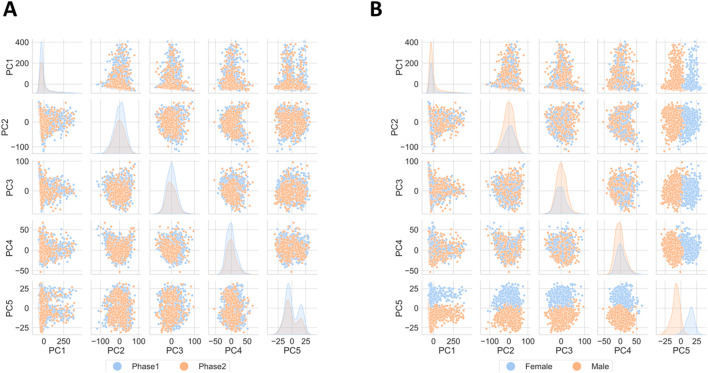
Principal Component Analysis results. **(A)** Depiction of variance with possible drivers like gender and **(B)** sequencing phase. No significant technical bias is detected, biologically based on gender, yet this driver accounts for a very small percentage of the overall variance.

The central subject of the exploratory data analysis was to ensure that the dataset does not include significant technical noise. This step was of particular importance, since the study conducted by the PPMI includes samples gathered from the same individuals (from both, control and case cohorts) over several visits over the course of 8 months, with each visit occurring 2 months from the previous one. Also, sequencing took place in two phases. Depictions of the percentual distribution of visits and age groups of individuals the samples of which were sequenced at distinct phases are presented within the image panel S1. Supplementary Figure S1 presents the distribution of age groups within each sequencing phase. Supplementary Figure S2 shows the distribution of samples grouped by visit across the sequencing phases where it is made apparent, that all samples taken on the second visit were sequenced during the second phase. The notion of separate sequencing phases as well as having samples from visits at different points in time, could potentially introduce artificial bias in the resulting expression values. Based on these variables Principal Component Analysis (PCA) was conducted as a measure to visualize the variance behavior and whether clusters based on the identified technical factors form. Figure 1A shows PCA results by gender, with PC5 (under 2% variance) indicating distinct clusters. Figure 1B shows the PCA results by sequencing phase, where no separate clusters form, thus the variance introduced by the sequencing phase may be deemed practically non-existent.

### 3.2 Differential gene expression analysis

**FIGURE 2:**
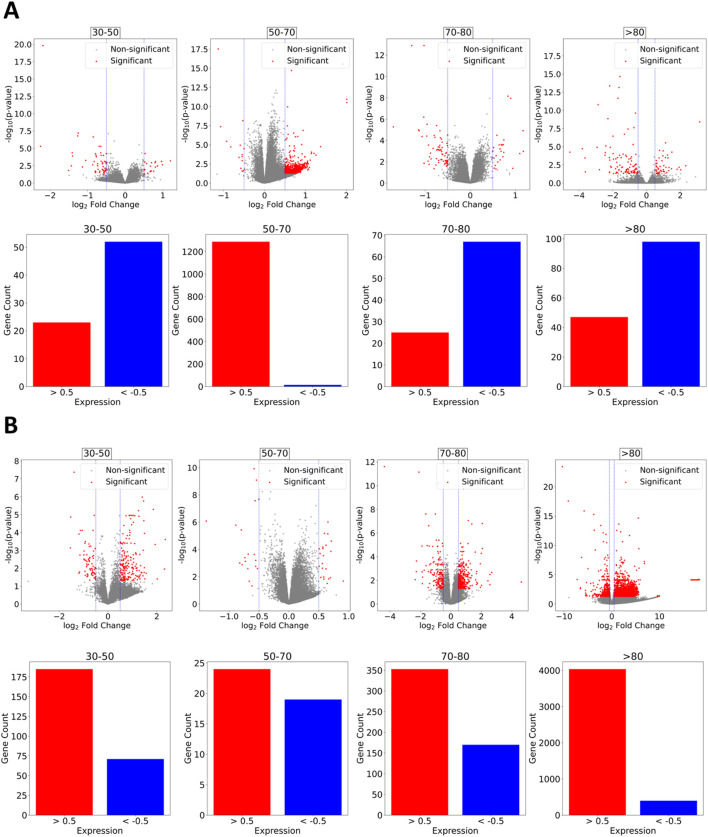
Differential Gene Expression. **(A)** Males. Overall trend across age groups towards downregulation with the exception of the age 50–70 group, which exhibits an extensive number of upregulated genes and relatively few downregulated ones. **(B)** Females. Expression motifs tend to upregulation. Significantly less downregulated genes compared to upregulated are observed for age groups 30–50 and over 80 years of age.

Differentially expressed genes were found to follow distinct expression patterns between males and females, where males displayed a more prominent trend to downregulation (Figure 2A) compared to females (Figure 2B), where most age groups had upregulated genes. Males between 30 and 50 years of age had about 50 downregulated genes and slightly over 20 upregulated. Age groups 70–80 and over 80 years of age displayed between 70 and 100 downregulated genes and 20 to 40 upregulated, respectively. The only difference in the overall expression pattern is displayed by the age group 50–70 years, where a vast amount of over 1,200 upregulated genes appears and only a few downregulated. We assessed whether the heterogeneity in expression patterns could be attributed to technical confounders by performing ANOVA on key metadata variables. The absence of statistically significant associations leads us to interpret this heterogeneity as an unbiased biological signal rather than a technical artifact.

Common genes across genders and among the same age groups were found to mostly have different expression patterns rather than similar ones. Downregulated genes for males and females aged 30–50 years are ENSG00000251652, LOC105374344, FOLR3 and CNTNAP3P2. Downregulation was similar for all except CNTNAP3P2, which showed a marked decrease compared to female expression. Age group 50–70 contained only 1 similar expression pattern for both, males and females, for gene RAP1GAP as a common downregulated one with a similar degree of expression and RNU1-4 as a common upregulated gene with almost identical expression. A comparatively significantly upregulated gene for females aged 70–80 was C4BPA. In the same age group, both males and females presented a downregulation of the gene NECTIN2, with the males having a higher degree of downregulation compared to females, whereas SFRP1 and OLFM1 were slightly more downregulated in females compared to males. Individuals older than 80 years of age appeared with a severely downregulated RNF182. NECTIN2 appeared again downregulated in males but significantly upregulated in females. Also, genes CLEC12A and CLEC12B presented distinct expression patterns for males and females with upregulation and severe downregulation, respectively.

### 3.3 Machine learning classification

**FIGURE 3:**
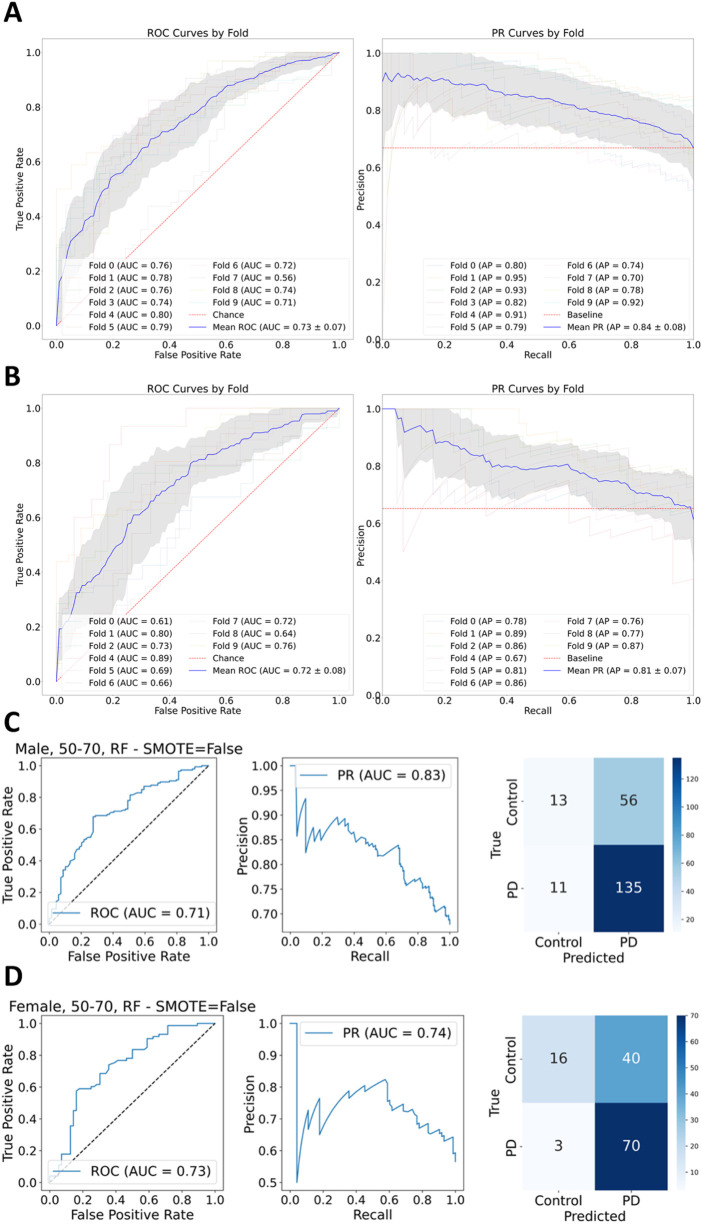
**(A)** XGBoost 10-fold cross validation ROC-AUC and PR-AUC display promising results across all folds for male stratum. **(B)** XGBoost 10-fold cross validation for female stratum presents likewise satisfactory results as ROC-AUC and PR-AUC suggest. **(C)** Predictions made by the Random Forest classifier for the male stratum present a ROC-AUC score of 0.71 and PR-AUC of 0.83, reflecting a moreover satisfactory result in favor of the case class. **(D)** Random Forest classification for the female stratum yields less satisfactory results compared to the performance on the male stratum in regards to the Precision-Recall, and a slightly better ROC-AUC score.

Machine learning was applied by employing the classifiers Logistic Regression, Support Vector Machine, Random Forest, and XGBoost. Training was performed on 80% of each stratum dataset while scikit’s (Buitinck et al., 2013) GroupShuffleSplit was applied to avoid overly optimistic prediction results because of feature leakage (Oosterhuis et al., 2024). The differentially expressed genes for each stratum were used as the feature set for training and testing the models. The goal of applying machine learning categorization was to find genes that mattered the most for telling apart health from disease and ultimately which genes might be potentially involved in the disease. Because of the moderate class imbalance (with a ratio of 2:1) presented across all strata, class weights were set to be balanced across all employed classifiers. For each stratum, the data were split into a training set (80%) and a hold-out test set (20%). Hyperparameter tuning was performed using 10-fold cross-validation on the training set to identify the optimal model. The performance of this selected model was then evaluated in two ways: first, via a 10-fold cross-validation on the entire stratum’s dataset, and second, via a final evaluation on the stratum’s hold-out test set. ROC-AUC and PR-AUC curves were generated for both the cross-validation (Supplementary Figures S3–S10) on the full dataset and the final test set evaluation (Supplementary Figures S11–S18). An exemplary depiction of the conducted 10-fold cross validation is presented for the XGBoost classifier in Figures 3A,B.

The metrics ROC-AUC as a function of the true positive predictions over the false positive ones and PR-AUC as a function of the precision over the sensitivity were calculated for each fold during ten-fold cross-validation. The means for ROC-AUC across all classifiers for predictions on the female stratum lie between the lowest value of 0.72 and the highest of 0.77, with the lowest value being accounted by XGBoost and the highest by Logistic Regression. PR-AUC lies between 0.81, returned by Random Forest and XGBoost, and the highest mean value of 0.83 returned by Logistic Regression and SVM. The best predictions for the male stratum were returned by XGBoost with 0.73 and 0.84 for ROC-AUC and PR-AUC respectively, while the lowest values were observed for Logistic Regression with scores for ROC-AUC as low as 0.55 and 0.7 for PR-AUC. The low performance of Logistic Regression on the male stratum is very close to a random guessing model, because of the proximity of the curve to the baseline of 0.5. XGBoost performs better on males than females due to the larger sample size, even though both groups have a 2:1 class imbalance ratio.

Table 1 summarizes precision and recall for each prediction class (HC = Healthy Control; PD = Parkinson’s Disease) as well as the ROC-AUC and PR-AUC scores for each classification model and gender stratum for ages 50–70 years. The scores captured for the Random Forest and SVM classifiers are high for both, males and females. XGBoost displays a high recall score for males and a lower one for females. Logistic Regression also provides a satisfactory recall with a recall score of 70%. In general, the metrics suggest a relatively modest performance regarding classification of true positives. Logistic Regression presents with a precision of over 70% for all genders. XGBoost and Random Forest deliver precision values of 74% and 70% correspondingly. Interestingly, the Random Forest model achieves its highest precision of 80% specifically for samples originating from female participants. The ROC-AUC and PR-AUC curves alongside with an appropriate confusion matrix were plotted and included in Supplementary Figures S11–S18. Despite the promising performance reflected during the validation phase of the XGBoost classifier, the categorization via Random Forest delivered a slightly better performance during prediction (Figures 3C,D).

**TABLE 1:** Detailed classification metrics - Values for precision and recall and ROC-AUC, PR-AUC for each classifier and gender (M = Male; F =Female) during prediction for age stratum 50–70 years of age.

		LR		SVM		RF		XGB	
		M	F	M	F	M	F	M	F
Precision	HC	0.37	0.56	0.52	0.68	0.50	**0.80**	0.59	0.59
	PD	**0.70**	**0.72**	**0.75**	0.69	**0.70**	0.65	**0.74**	0.68
Recall	HC	0.38	**0.71**	0.45	0.54	0.13	0.36	0.33	0.57
	PD	**0.70**	0.58	**0.80**	**0.81**	**0.94**	**0.93**	**0.89**	**0.70**
ROC-AUC		0.582	0.707	0.641	0.689	0.714	0.732	0.694	0.707
PR-AUC		0.739	0.747	0.790	0.703	0.828	0.737	0.790	0.743

Scores above 70% appear in bold.

**FIGURE 4:**
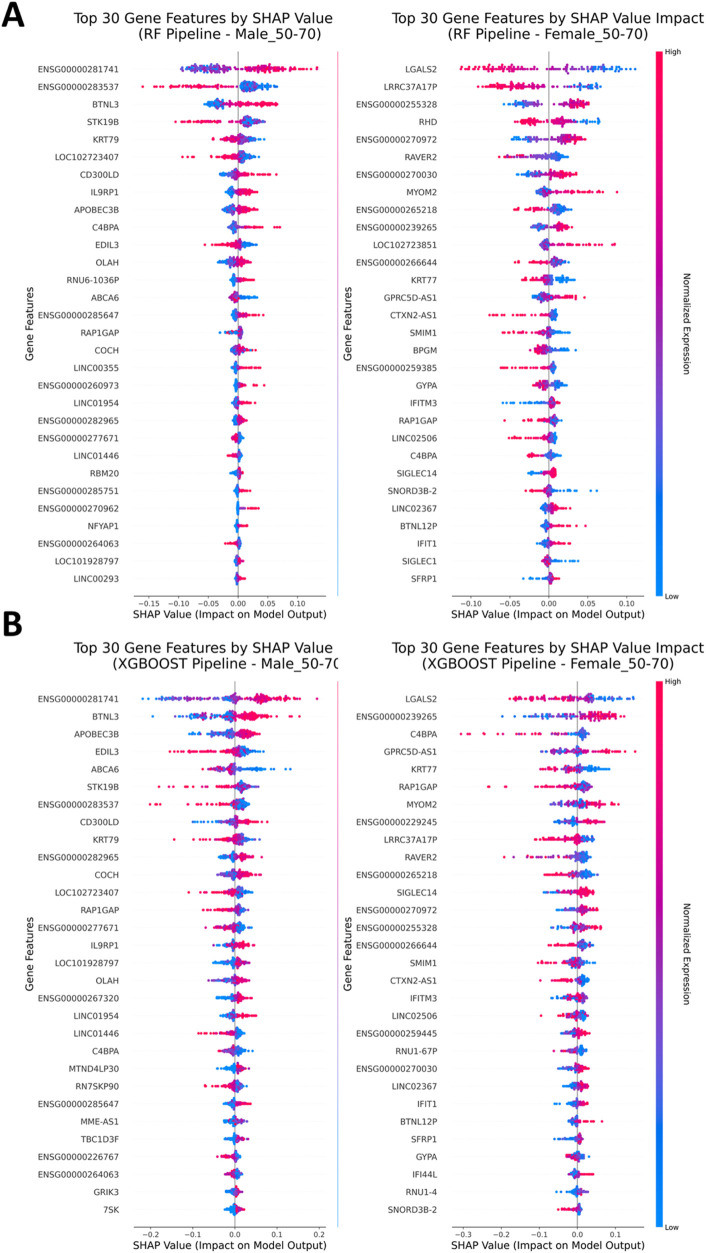
SHAP Analysis. **(A)** SHAP Analysis results after assessment of the Random Forest classifier (Left: Males, Right: Females). Substantial differences in gene importance during classification can be observed. **(B)** SHAP Analysis for the XGBoost classification model shares similarities in gene importances as well as expression motifs with the Random Forest classifier in the set of the most impactful genes in the top-half positions of the plots among the respective strata.

As a means to extract information about which genes were deemed important by the classification models and thus influenced the prediction, as well as the extent, SHAP analysis was performed for each classification model (Lundberg and Lee, 2017). SHAP analysis offers a clear and straightforward method for illustrating feature importances by capturing both, the influence of specific features on decision making and whether a feature has an impact on negative or positive categorization based on its expression values (Supplementary Figures S19–S22). Generally, the features that influenced classification the most for males and females are different. This could support a hypothesis of a distinct transcription motif for Parkinson’s disease in males and females and consequently the involvement of different mediating pathways in disease pathogenesis. Accordingly, the expression patterns for the same gender stratum, as derived from SHAP analysis, align across the models. For the classification models Logistic Regression and SVM the common genes LFALS2 and LRRC37A17P present with a high score in regard to classification importance which also align by having a similar expression motif. Similar motifs are further presented by the Random Forest classifier for the same gender stratum (Figure 4A). In the male stratum, the genes ENSG00000283537, STK19B and KRT79 display importance with downregulated motif in favor of the case cohort while, on the other hand, genes IL9RP1, ENSG00000281741 and BTNL3 present as classification drivers for the case cohort with an upregulated motif. The XGBoost classifier presents LGALS2 again as an important feature for females, yet the gene LRRC37A17P is not included within the three top-most ranked features. Instead, the higher ranks are occupied by the genes ENSG00000239265, C4BPA and GPRC5D-AS1 which were deemed as low-importance features by the other classification models (Figure 4B). For males, the Random Forest and XGBoost classifiers display similarities among the higher ranked features, in particular, ENSG00000281741, BTNL3 and STK19B. Conclusively, SHAP analysis showed similar results among the gender strata within the same age group, while the similarity is not merely justified by the placement of the genes among the ranks but also by the similarity in expression motifs.

### 3.4 Functional enrichment analysis

The set of differentially expressed genes was imported into Cytoscape and the respective networks were constructed. Functional enrichment analysis and publication enrichment were performed by leveraging Cytoscape’s built-in connectivity with the STRING database (Szklarczyk et al., 2023). The results retrieved for females were dominated by the genes IFITM3, SIGLEC1 and MYOM2 which formed a network as presented in Supplementary Figure S23. Darker colored nodes represent higher absolute expression values whereas lighter ones represent lower. The main characteristic in this network is the presence of interferons, which signify immune response to inflammation or even cellular damage (Kopitar-Jerala, 2017). Publication enrichment via the STRING database delivered results for a subnetwork composed of genes RSAD2, IFIT1, SIGLEC1 and IFI44L (Supplementary Figure S23) which are linked to the Janus kinase (Yamaoka et al., 2004) and involved in the Jak/Stat pathway, which has been linked to Parkinson’s disease as a potential therapeutic target (Lashgari et al., 2021) and neuronal degeneration (Cooray et al., 2023). The summarized results as delivered from the STRING database are presented in Supplementary Table S1 for the functional enrichment and in Table 2 for publication enrichment.

**TABLE 2:** Publication enrichment excerpt from the STRING database: Results for females 50–70 years of age contain pathways related to janus kinase and neuroinflammation.

Description	FDR	PMID
Janus Kinase Inhibitors in the Treatment of Type I Interferonopathies: A Case Series From a Single Center in China	1.75 × 10^−5^	PMID:35418997
JAK inhibitors: a potential treatment for JDM in the context of the role of interferon-driven pathology	7.98 × 10^−5^	PMID:34563217
Neuroinflammation, autoinflammation, splenomegaly and anemia caused by bi-allelic mutations in IRAK4	1.2 × 10^−4^	PMID:37744344

The enrichment results retrieved for the differentially expressed genes via the Enrichr API do not deviate much from the STRING results, as the enrichment terms with the highest statistical significance concern interferon transcription and indicate ties to the immune system. Additionally, to gene ontology terms, transcription factor databases were consulted as well, which also yield immune-related enrichment results. Interesting is the finding of the transcription factor HESX1, for which a search on the Gene4PD database (Li et al., 2021) returned results from the Humap Phenotype Ontology (Gargano et al., 2024) referring to motor issues, tremors, hyposmia and anosmia (Jankovic and Tan, 2020; Mitchell et al., 2025) and pathological levels of prolactin. The latter has been a subject of research, yet an involvement in Parkinson’s disease pathology could not be conclusively confirmed (Al-Kuraishy et al., 2023).

The results for the male stratum are dominated by the presence of keratin. According to the results from differential gene expression analysis, the gene KRT77 is downregulated and was also involved as one of the 30 topmost important features in machine learning classification, according to the results from SHAP analysis. The results from the analysis conducted via Cytoscape on the other hand do not align with this finding, since there are several keratin-like proteins presenting as upregulated in the respective network as depicted in Supplementary Figure S24. Nevertheless, the impact of keratin in Parkinson’s disease is discussed in publications (Wang et al., 2022; Liu et al., 2025). In the absence of compelling and conclusive evidence supporting a biological role for keratin in PD pathogenesis, we interpret this finding as a potential artifact. Given the lack of an established mechanistic link, we consider it more likely to stem from an unidentified technical bias or confounding factor than to represent a genuine biomarker. Unlike with the results for the female stratum, there were not enrichment terms returned related to the immune system. Enrichment for publications did not yield any results for the male stratum, while functional enrichment was unsurprisingly dominated by enrichment terms related to biological processes related to keratin while genes like LCE1A, LC5AA repeatedly appear in the respective process (Supplementary Table S2). The dominance of keratin, as provided by the results from network analysis, could be related to the fact that keratin degradation is regulated by the ubiquitin-proteasome pathway, which is also linked to PD pathogenesis (Lim and Tan, 2007). While the reported fold changes are statistically significant, their magnitude could partially be attributed to covariate-driven overamplification and/or low-level sample contamination. For instance, ambiguously expressed transcripts near detection thresholds might appear artificially inflated. Future studies with stratified sampling and RNA-seq verification could clarify whether these signals reflect biological variation or methodological artifacts.

Functional enrichment was conducted for the emerged DEGs via the Enrichr API, which did not yield any statistically significant results, with the adjusted p-value being either very close or equal 1. Despite the low statistical score for the enrichment results, the enrichment terms bear in parts a proximity to processes and pathways involved in Parkinson’s disease (The results are summarized in Supplementary Table S3). The first four entries reflect cellular components and biological processes of the nervous system. The relationship of the entry IL-2/STAT5 signaling concerns processes of the immune system. The entry Xenobiotic Metabolism corresponds to the metabolism of foreign chemicals that may impact metabolic processes and, as a general term, may refer to chemicals like pesticides as well as other drugs (Croom, 2012). The relationships between pesticides and Parkinson’s disease in respect to influences to xenobiotic metabolism have been analyzed in the past (Le Couteur et al., 1999). A potential association of pathways related to lipid metabolism and Parkinson’s disease is analyzed by Alecu and Bennett, 2019. The possible manifold influence of heme metabolism on neurodegenerative diseases is described by Chiabrando et al., 2018. The entries that follow are linked to immune responses to viral pathogens like SARS CoV2 as well as post-COVID neuroinflammation and chronic oxidative stress. Enrichment for gene TUBB8 is related to Parkin and Ubiquitin pathways, with a potential role in protein degradation disorders that are linked to SYNCA accumulation and thus to neuronal degeneration in Parkinson’s disease (Zhao et al., 2024). Copper homeostasis and its involvement in cell signaling is also in alliance with Gaggelli et al., 2006, where links to neurodegenerative disorders are explored and biochemical correlations between copper ions and SYNCA are analyzed. In the last three entries, Alzheimer’s disease is mentioned and finally, the enrichment term that sets a link between TUBB8 and Parkinson’s disease. Genetic variants implicated in PD pathogenesis have also been documented, based on analyses of neurons derived from the substantia nigra of individuals with Parkinson’s disease (Simunovic et al., 2010).

## 4 Conclusion

The present work reveals significant differences in gene expression between male and female pathological samples within the same age group (50–70 years). Functional network analysis, based on differentially expressed genes identified through stratified analysis, revealed immune-related signatures, while male samples exhibited a significant enrichment of keratin proteins. The enrichment analysis indicates that whole blood may harbor transcriptomic signatures associated with PD. Despite substantial existing evidence, the assumption lacks a definitive and distinguishable connection to established biological processes in PD—such as those implicated in protein degradation pathways. The strong difference in expression patterns among male and female as well as the differential expression and sequence of important features, which emerged from the SHAP analysis, allows us to understand that the disease exhibits sex-specific biological expression. There is also evidence suggesting shared biological pathways that contribute to the disease’s pathogenesis.

The limitations of this work primarily focus on the lack of further datasets for validating the performance of the established models. The reason behind this omission is the scarcity of immediately available and suitable datasets, that were created using the same sequencing platform. The authors consciously abstained from using microarray-based datasets or high-throughput ones that originate from a different platform and the demanding and error-prone pre-processing involved by choosing a different validation set. Also, the performance of the employed models presumably leaves room for improvement. A possible and direct optimization would affect the hyperparameter tuning, considering a more strategic approach. Apart from an immediate change on model parametrization, different normalization or variance stabilizing actions would probably yield improvement but also a different stratification strategy. Furthermore, the results for the male stratum require rigorous validation, since there is a serious lack of undisputable evidence that would reliably link Parkinson’s disease pathogenesis with Keratin related pathways. Nevertheless, the present study positions itself among a plethora of publications, that implement efforts to aid the discovery of biomarker detection and the mapping of functional networks in Parkinson’s disease. Yet, there are few that use blood-based samples, as, for example, the work of Shamir et al., 2017, where an SVM model was created with classification performance similar to the one established in this paper. Tabashum et al., 2024 systematically analyzed the landscape of machine learning models for Parkinson’s disease, by assessing a considerable number of publications by several parameters, like parametrization, validation strategy, sampling strategy and sample sources. Given the information conveyed by the work of Tabashum et al., we consider the major strength of the present work lies in the demographic stratification, the employment of multiple classification models, the use of an easily accessible source of sample biospecimen and the respective assessment of SHAP analysis to elucidate model decision and potential revelation of candidate biomarkers.

## Data Availability

Publicly available datasets were analyzed in this study. All RNA-Seq data and metadata used for the analysis presented by this study are available for downloading from PPMI (https://www.ppmi-info.org/access-data-specimens/download-data) through Laboratory of Neuro Imaging (LONI) Image Data Archive (IDA).
