# Supplementary material for: Computational analysis of transcriptome data and mapping of functional networks in Parkinson’s disease
Source: Front Bioinform. 2025 Nov 19;5:1690229. doi: 10.3389/fbinf.2025.1690229 (PMC12672545; doi:10.3389/fbinf.2025.1690229)
Supplement: Supplementary file 1 [file Supplementaryfile1.docx]

Supplementary Material

Computational Analysis of Transcriptome Data and Mapping of Functional Networks in Parkinson’s Disease


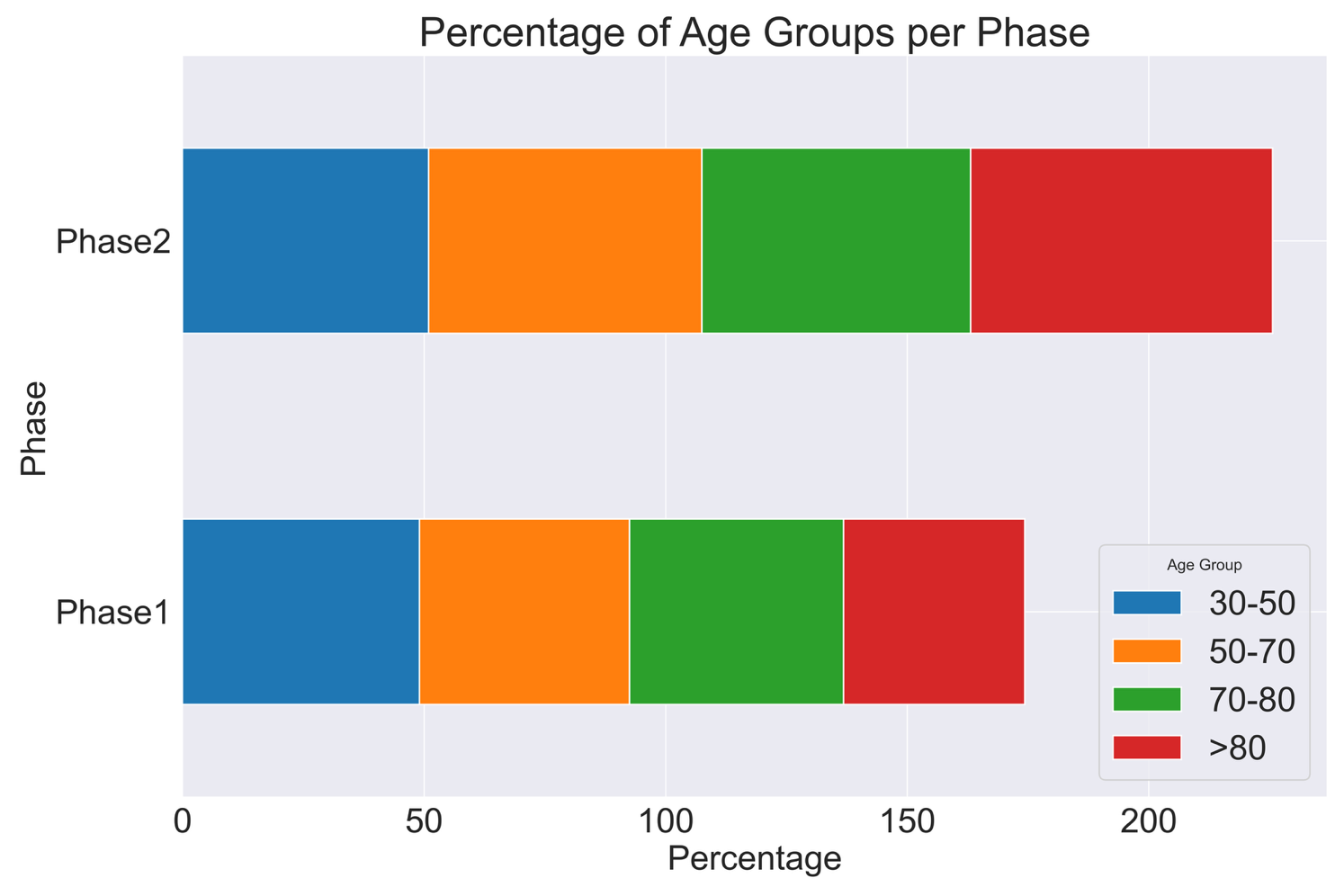


Figure S1: Distribution of age groups per phase show that both phases contain samples from individuals of all age groups as those were created during stratification


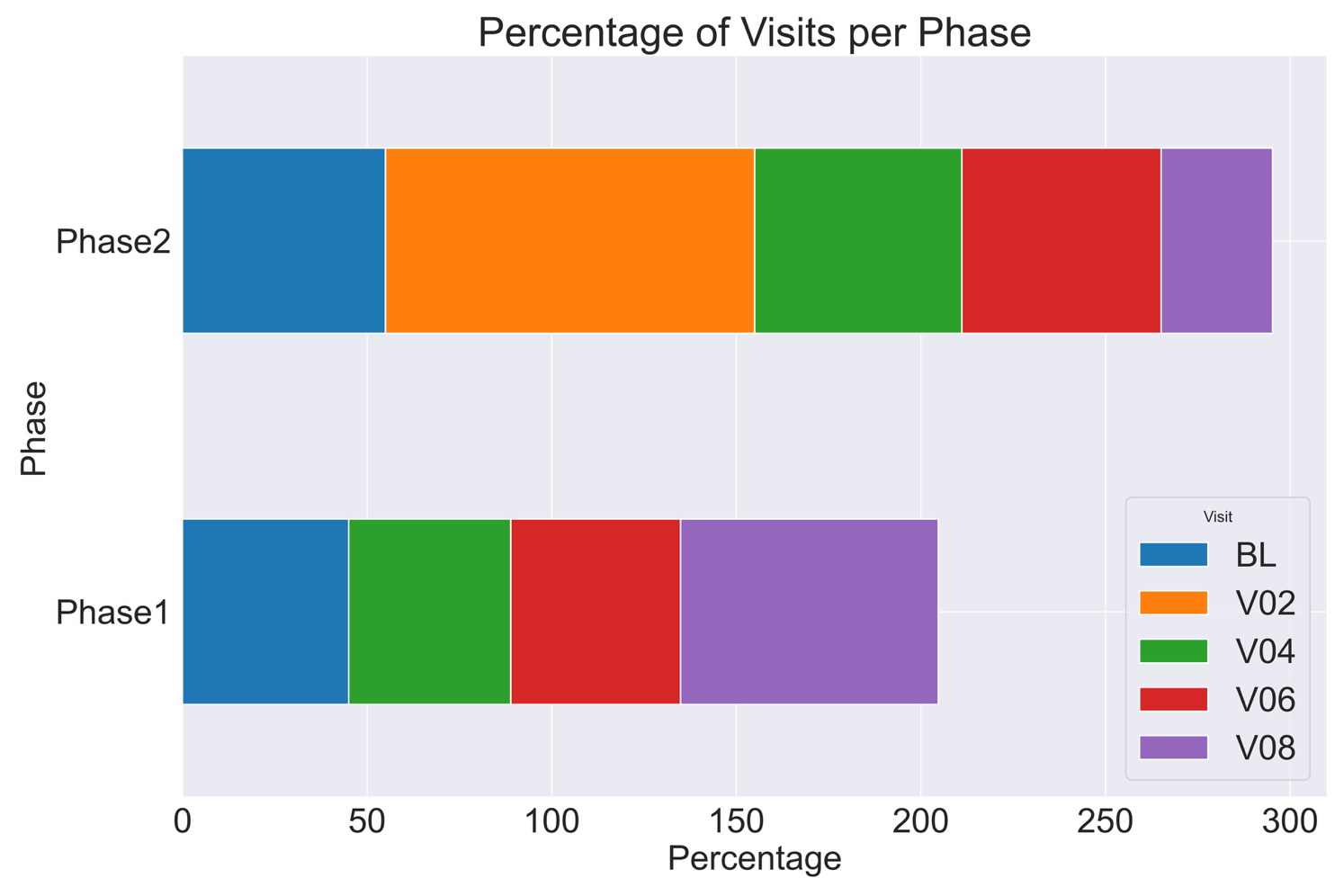


Figure S2: The proportion of samples from each visit and the phase those were sequenced in. Samples from visit V02, the first visit after the baseline one, were sequenced exclusively during the 2nd phase


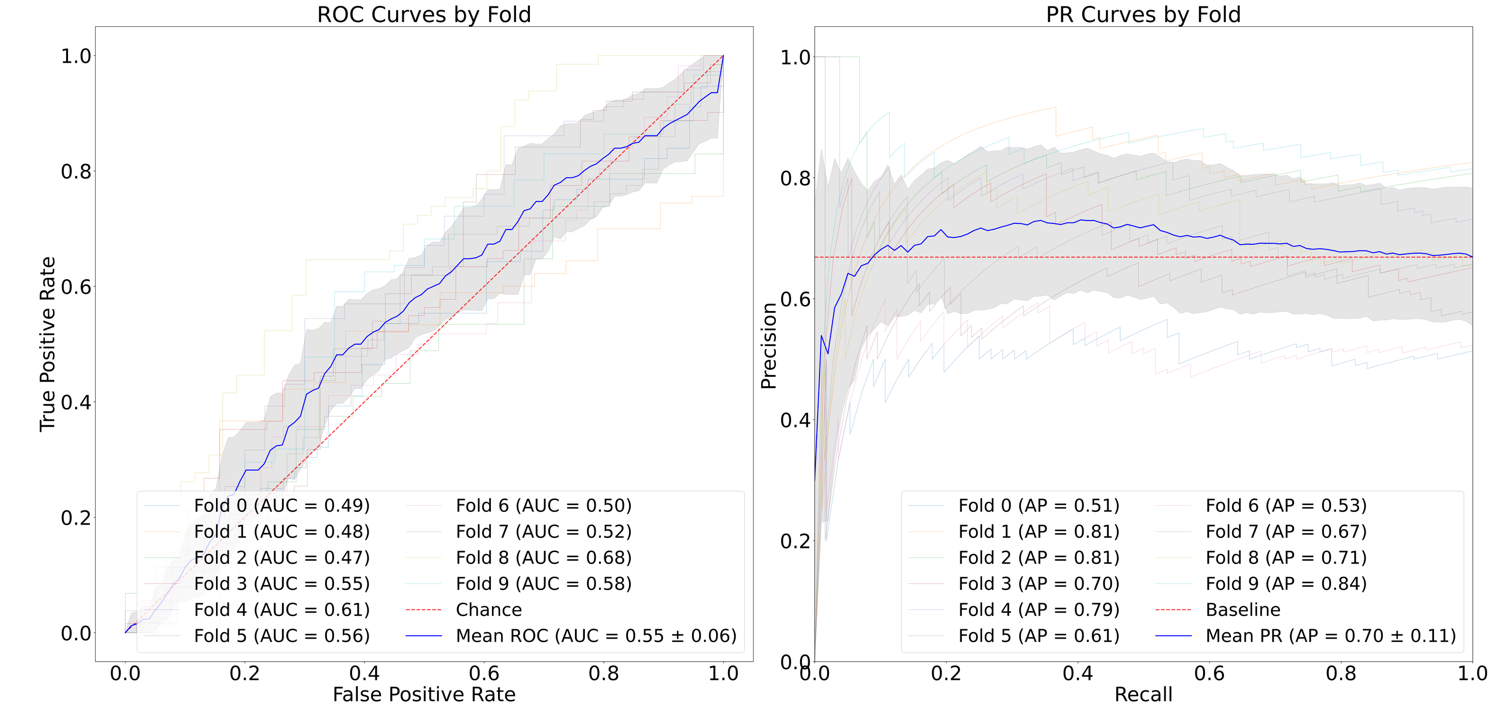


Figure S3: Logistic Regression k-Fold Cross Validation Males


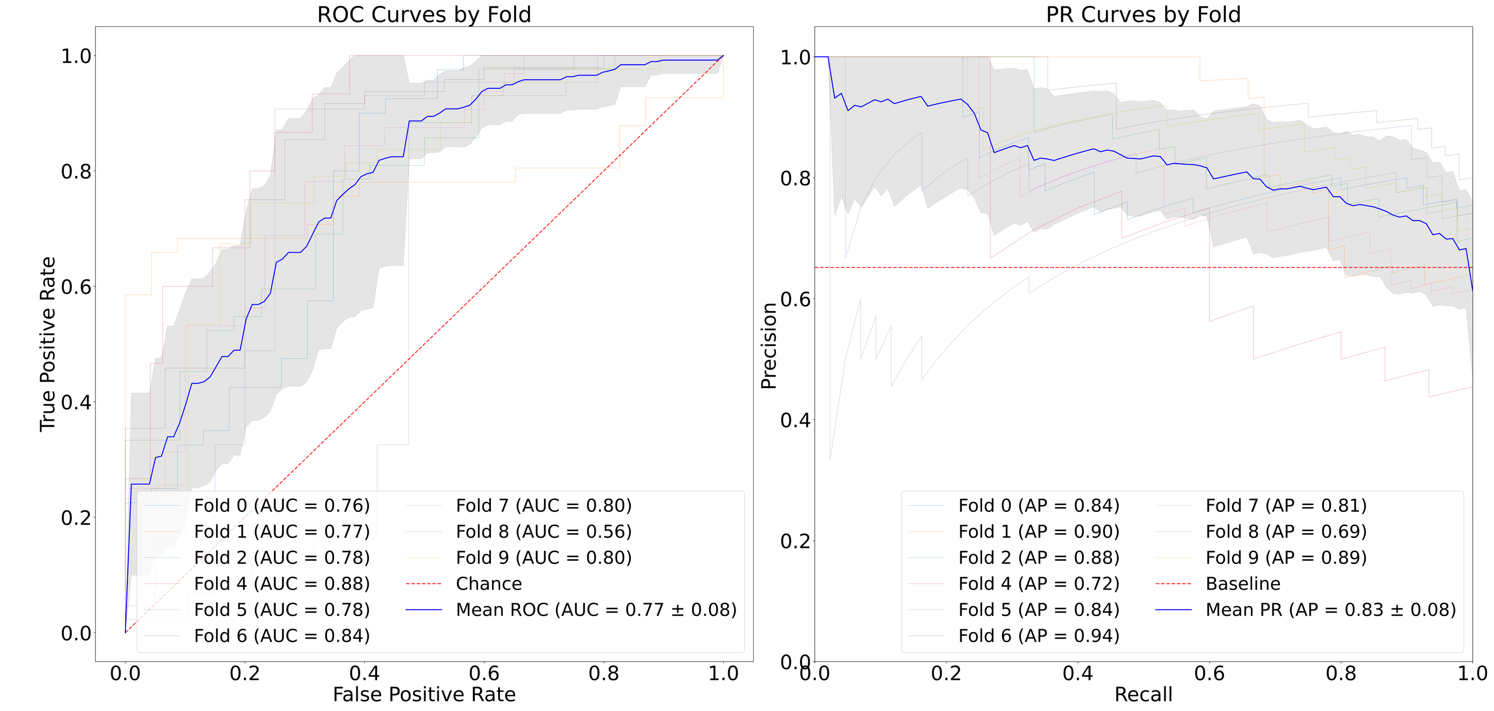


Figure S4: Logistic Regression k-Fold Cross Validation Females


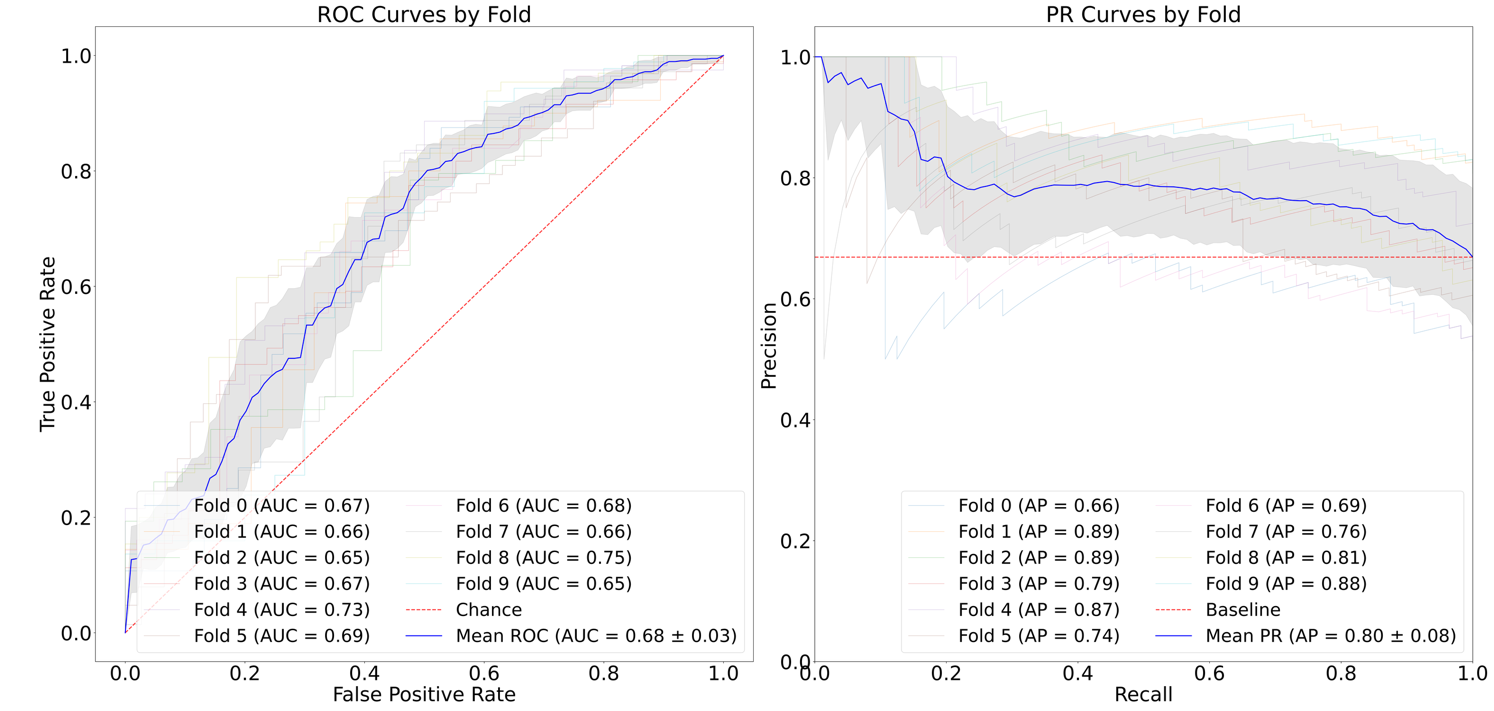


Figure S5: SVM k-Fold Cross Validation Males


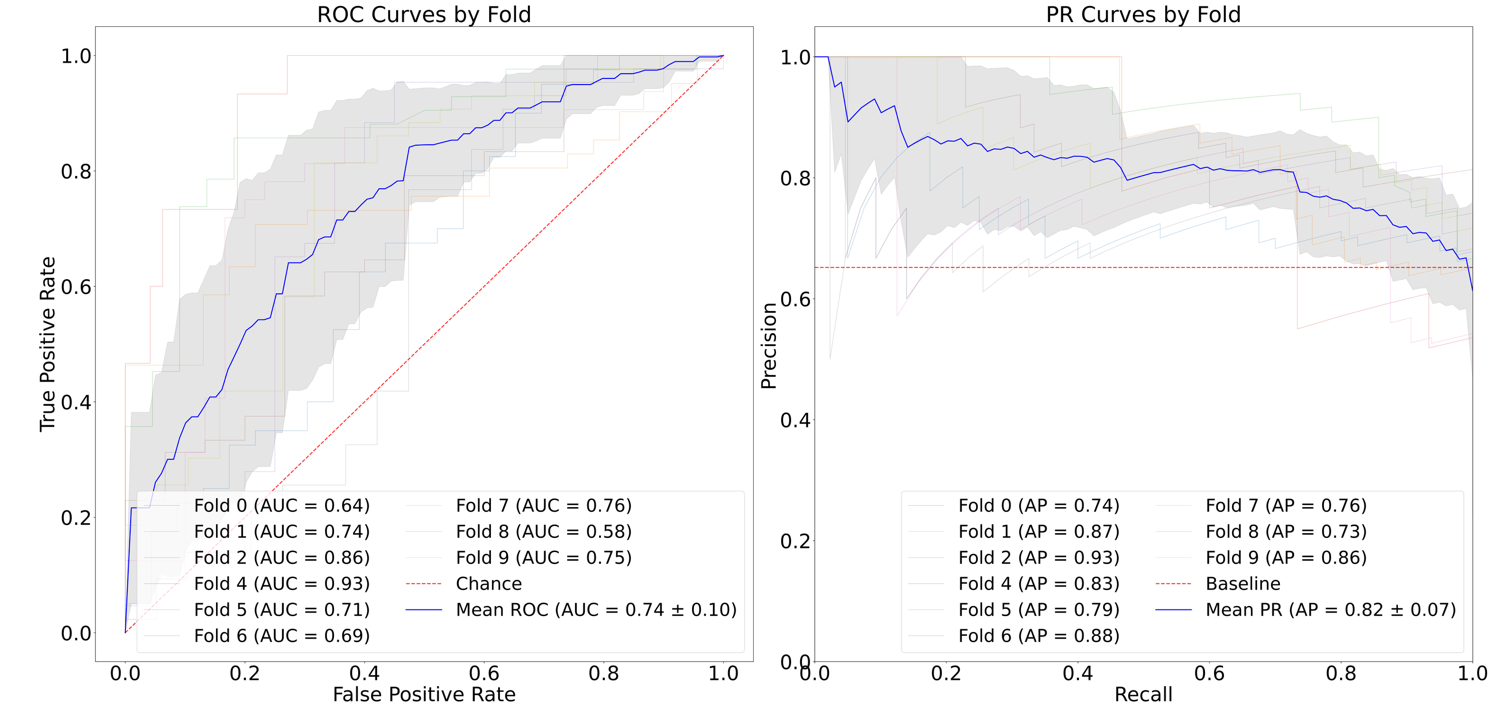


Figure S6: SVM k-Fold Cross Validation Females


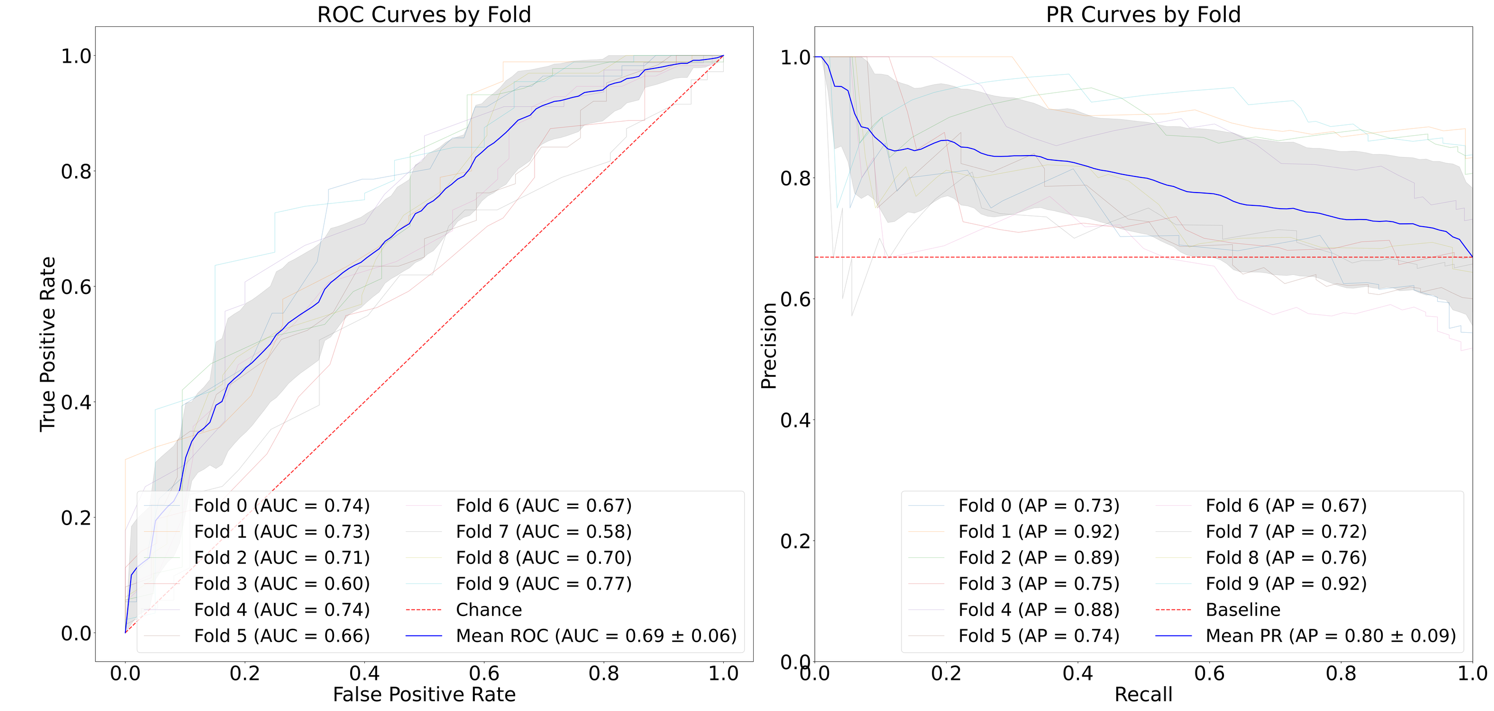


Figure S7: Random Forest k-Fold Cross Validation Males


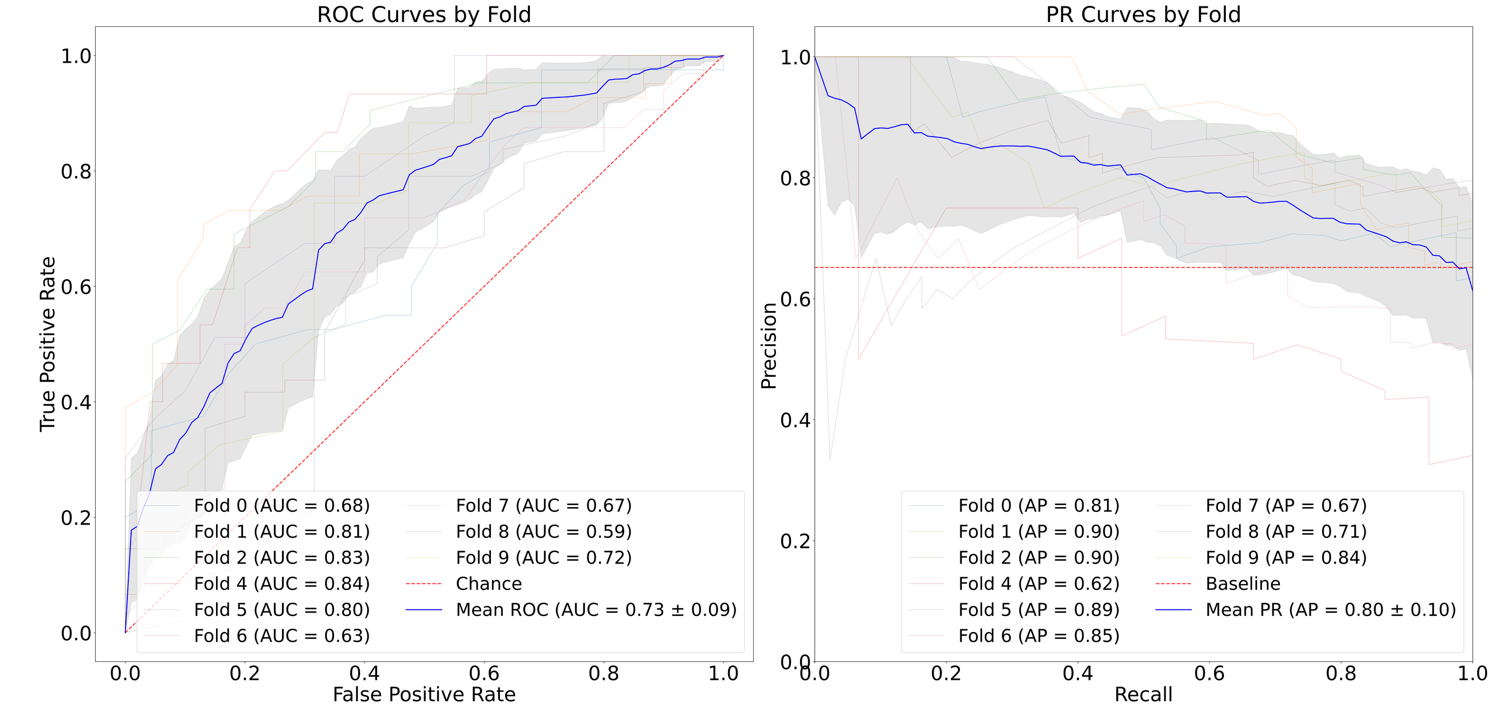


Figure S8: Random Forest k-Fold Cross Validation Females


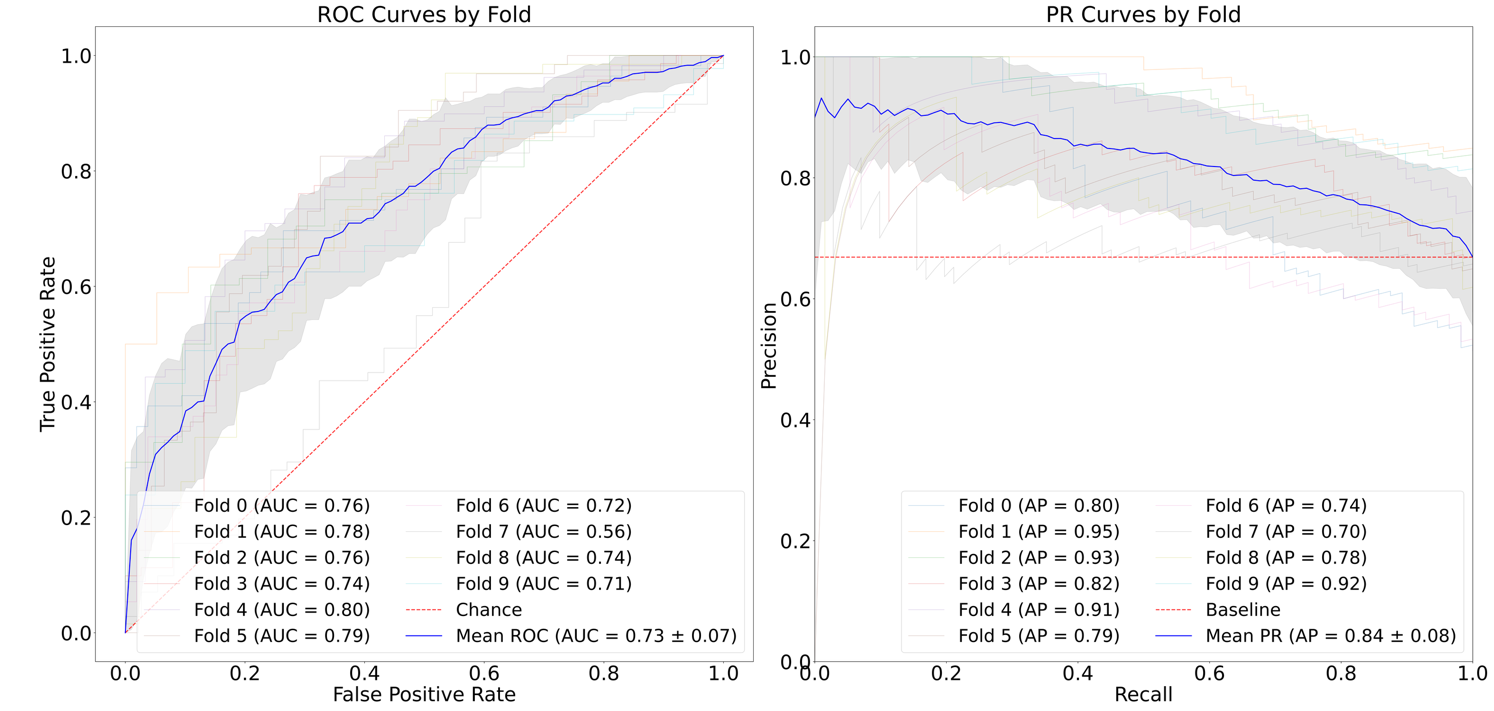


Figure S9: XGBoost k-Fold Cross Validation Males


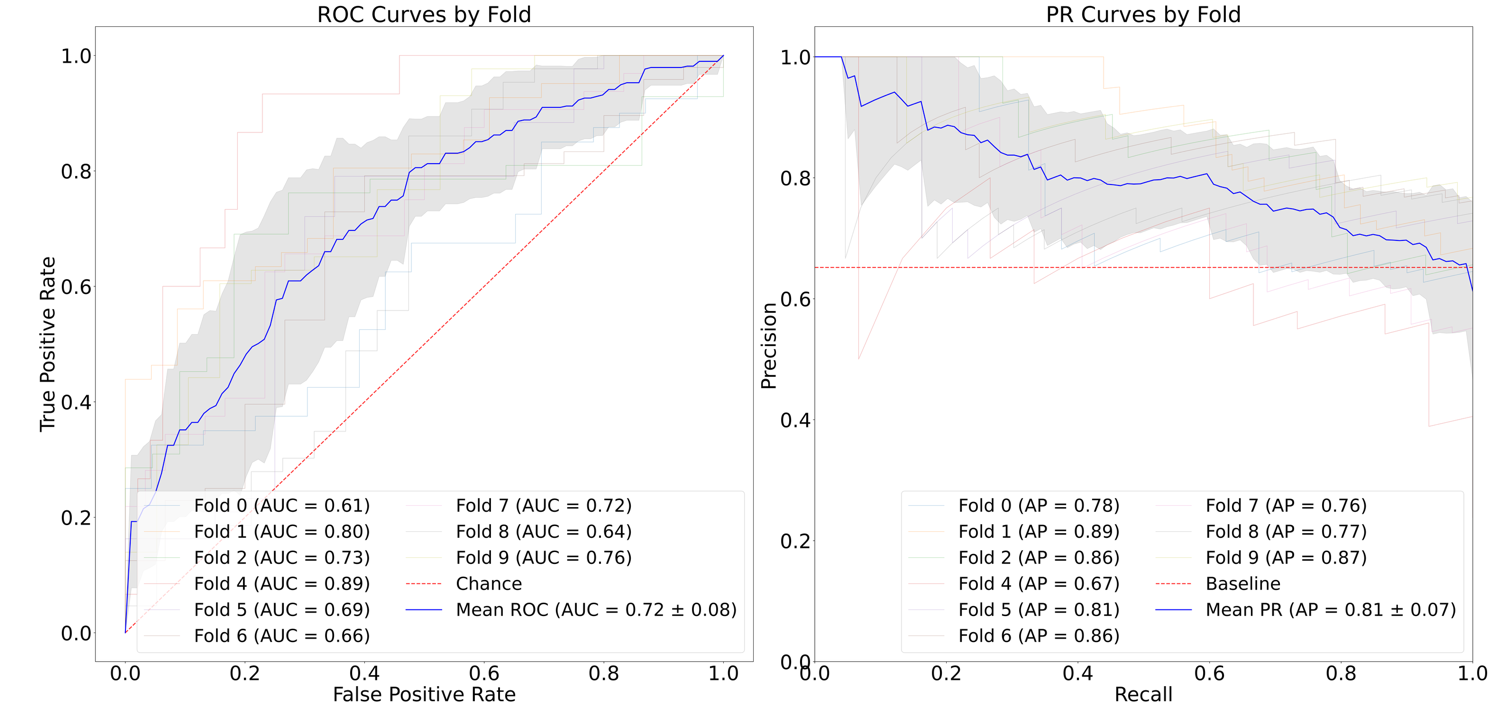


Figure S10: XGBoost k-Fold Cross Validation Females


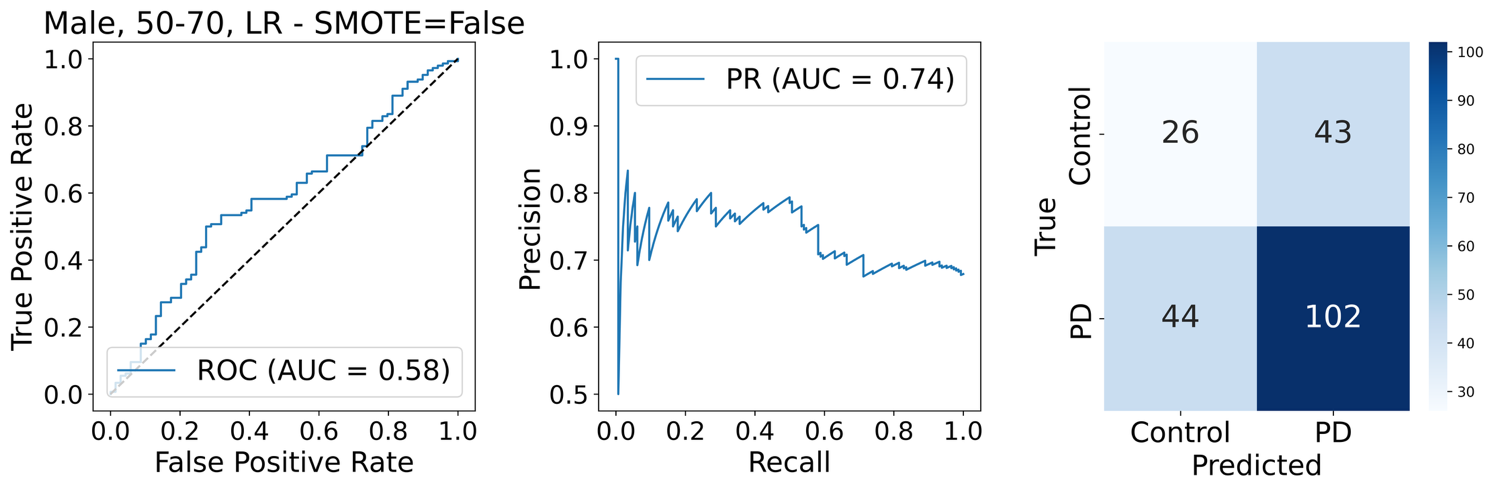


Figure S11: Logistic Regression predictions – Males


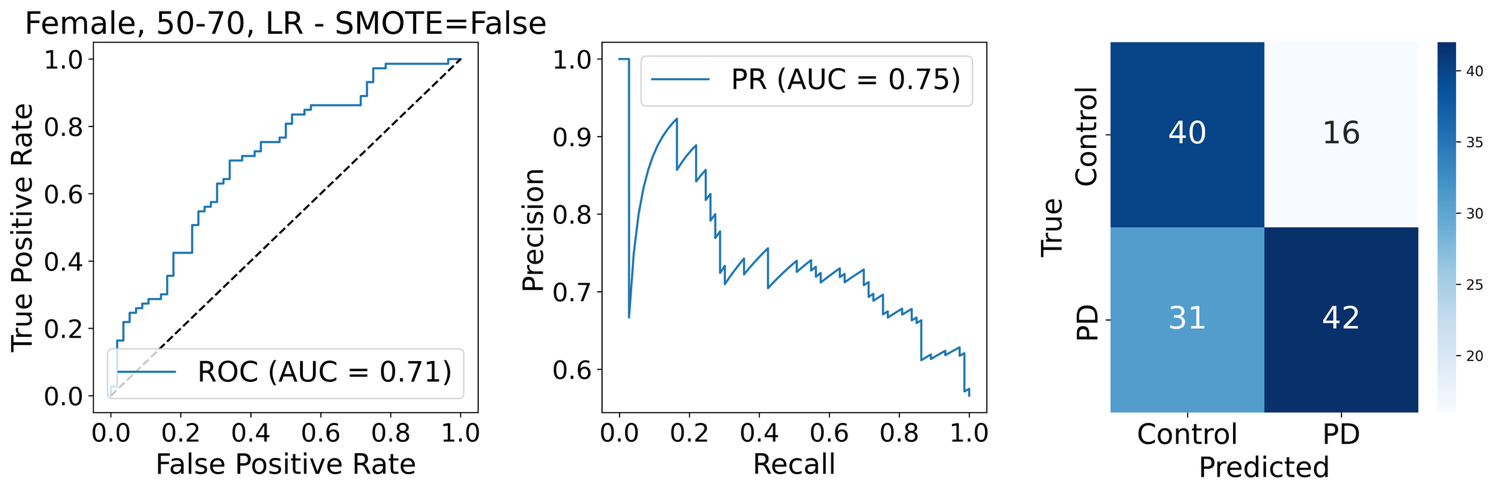


Figure S12: Logistic Regression predictions – Females


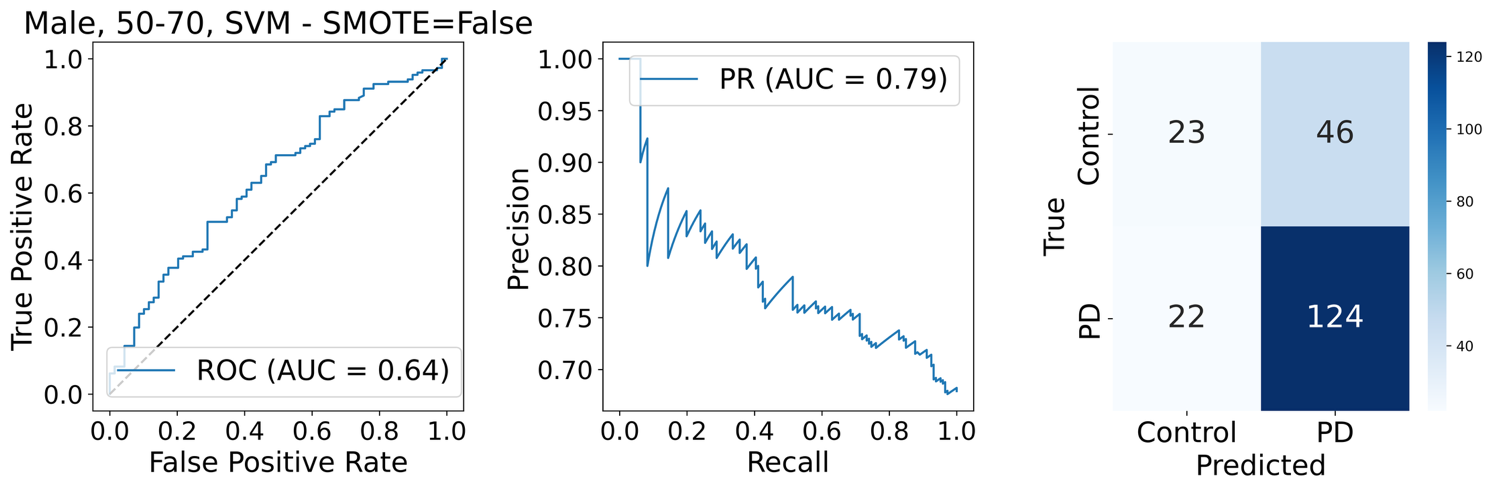


Figure S13: SVM predictions – Males


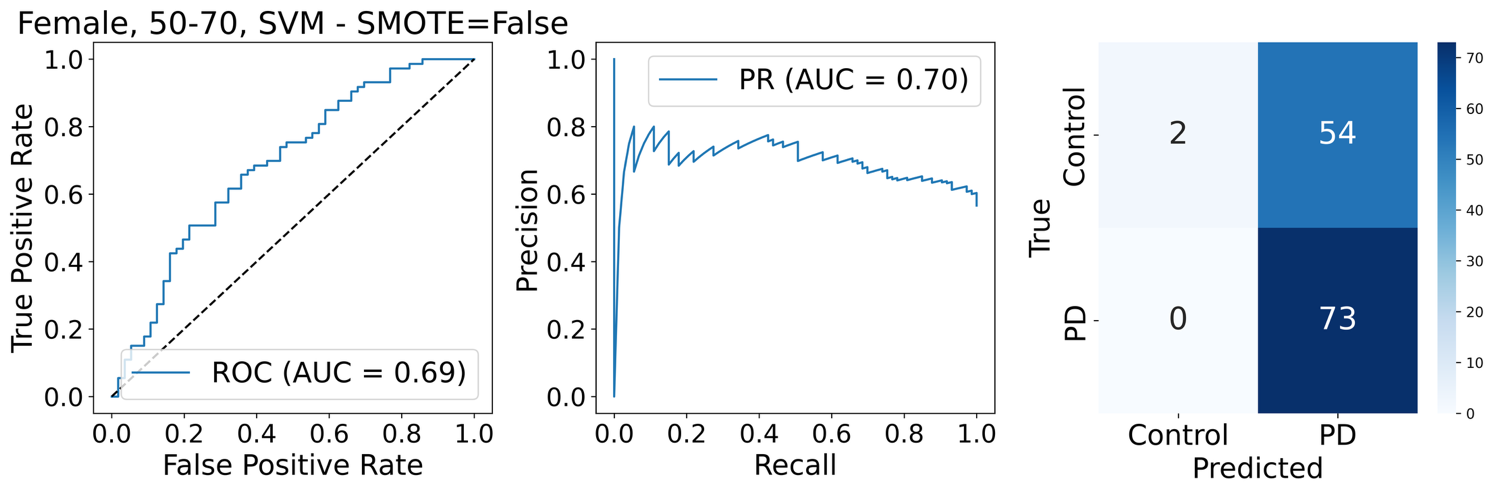


Figure S14: SVM predictions – Females


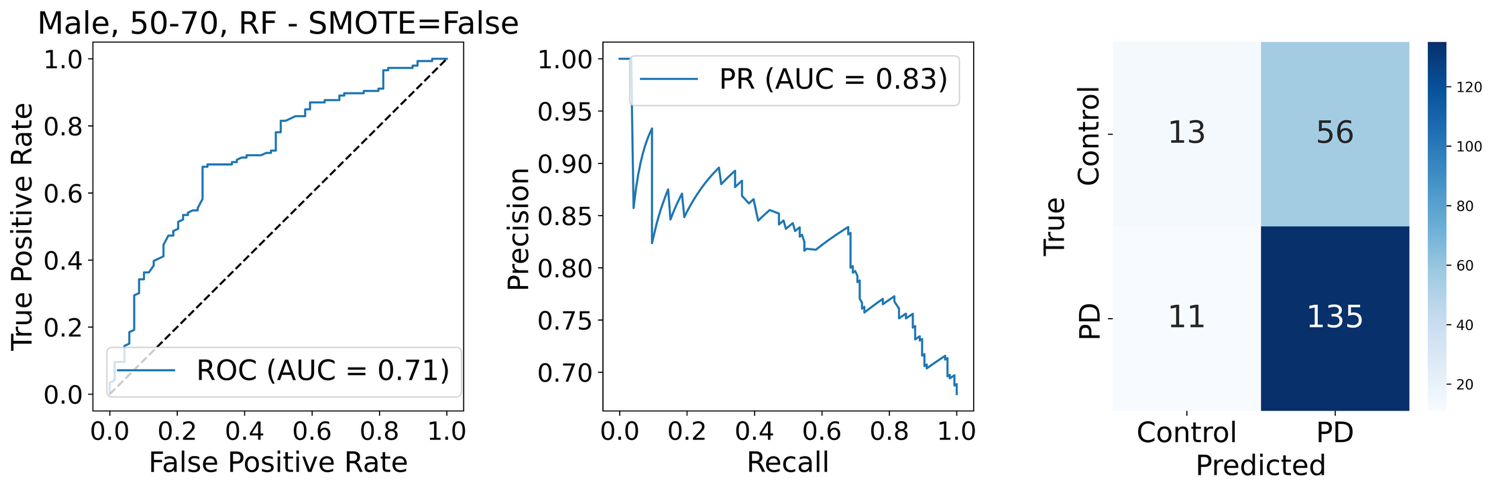


Figure S15: Random Forest predictions – Males


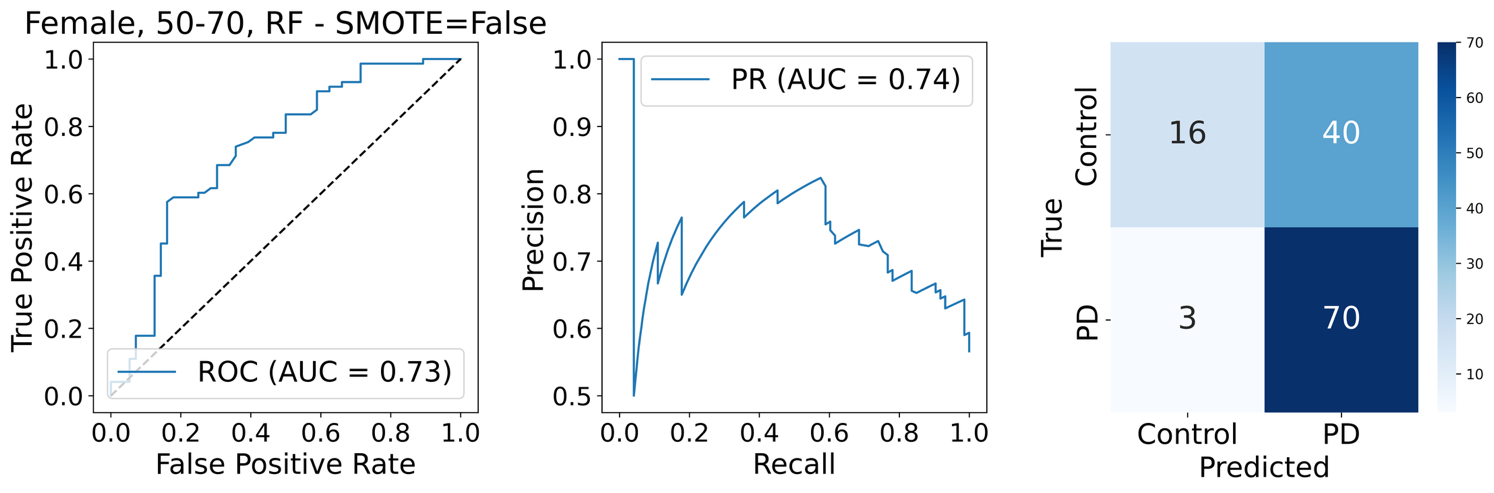


Figure S16: Random Forest predictions – Females


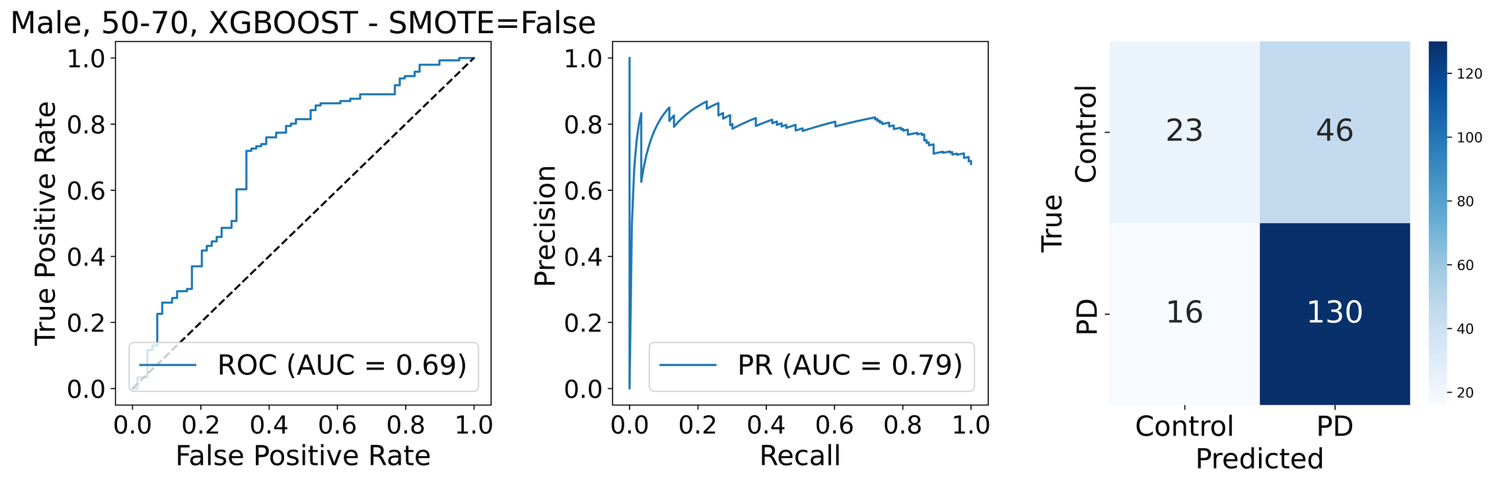


Figure S17: XGBoost predictions – Males


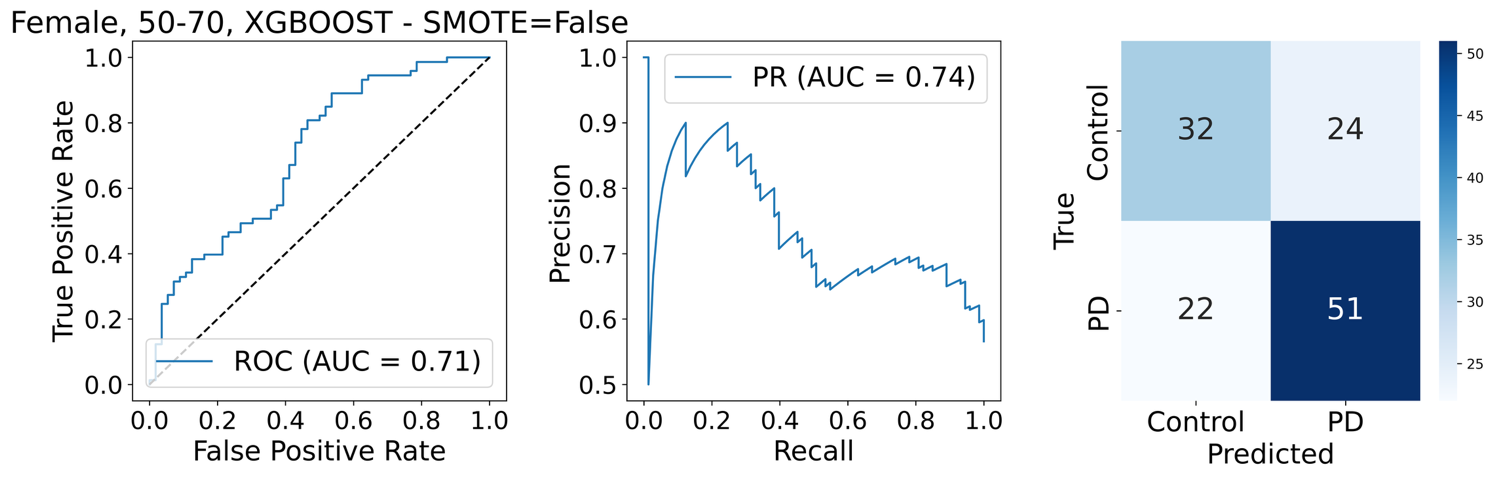


Figure S18: XGBoost predictions – Females


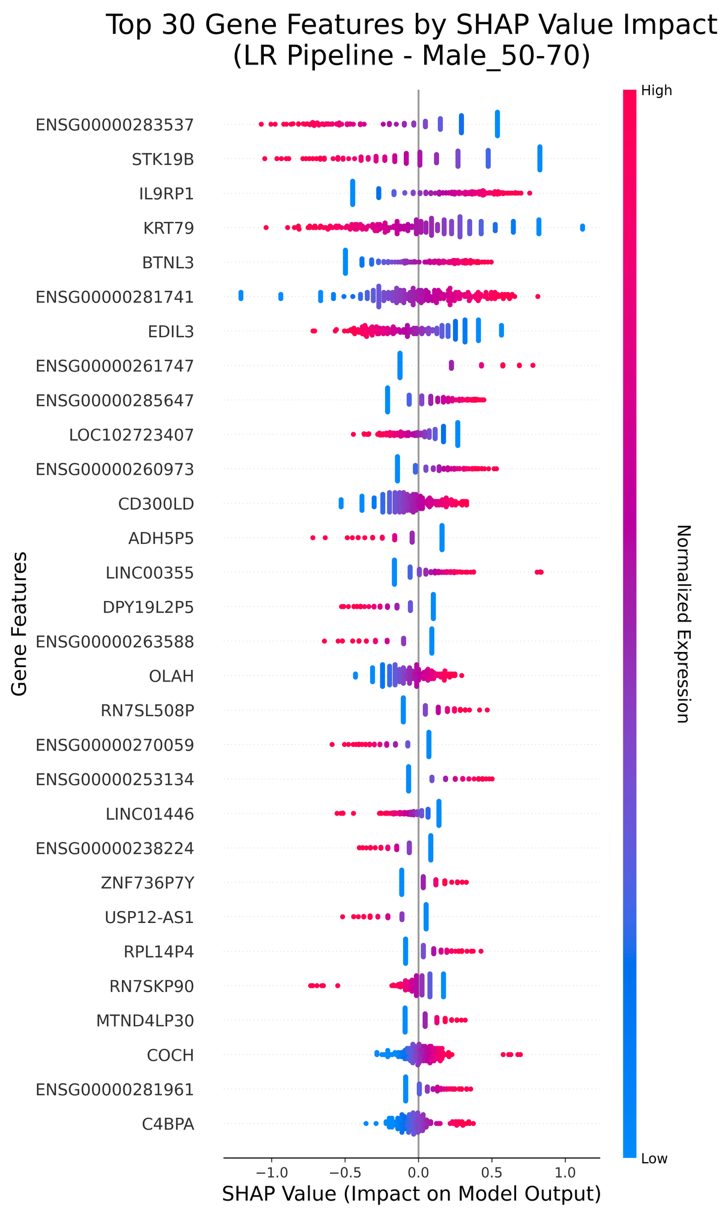

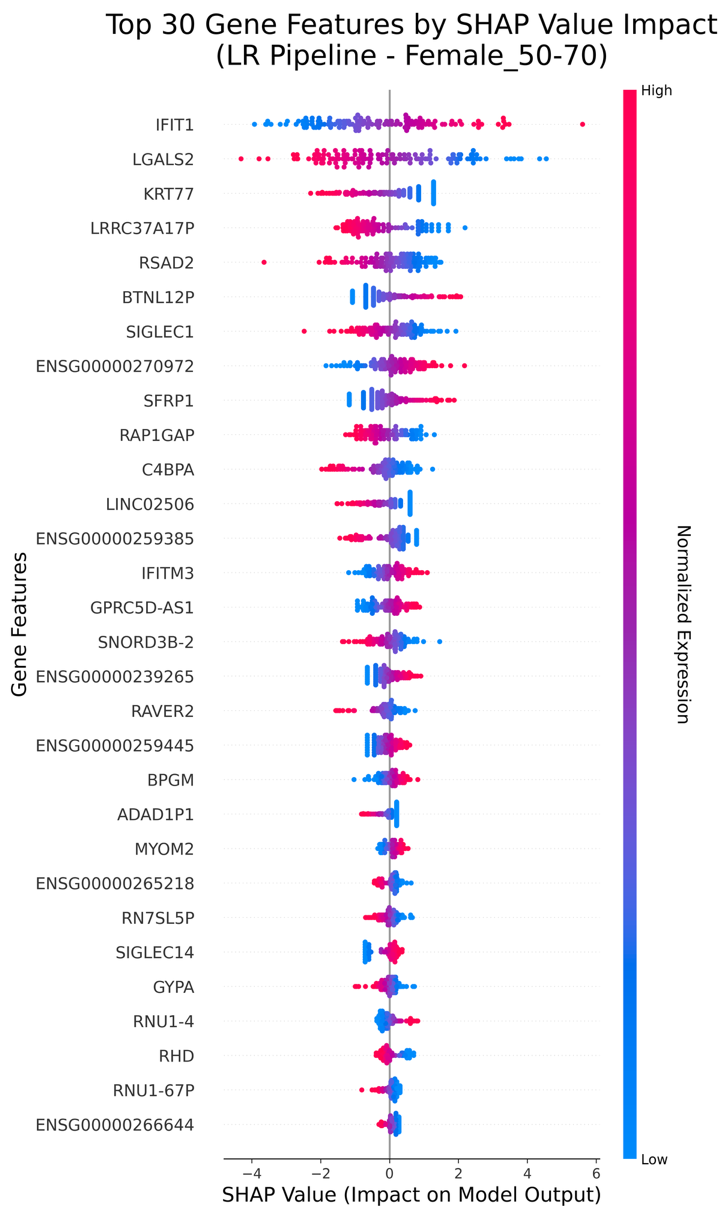


A: Males 50-70 years of age

B: Females 50-70 years of age

Figure S19: SHAP Analysis Logistic Regression


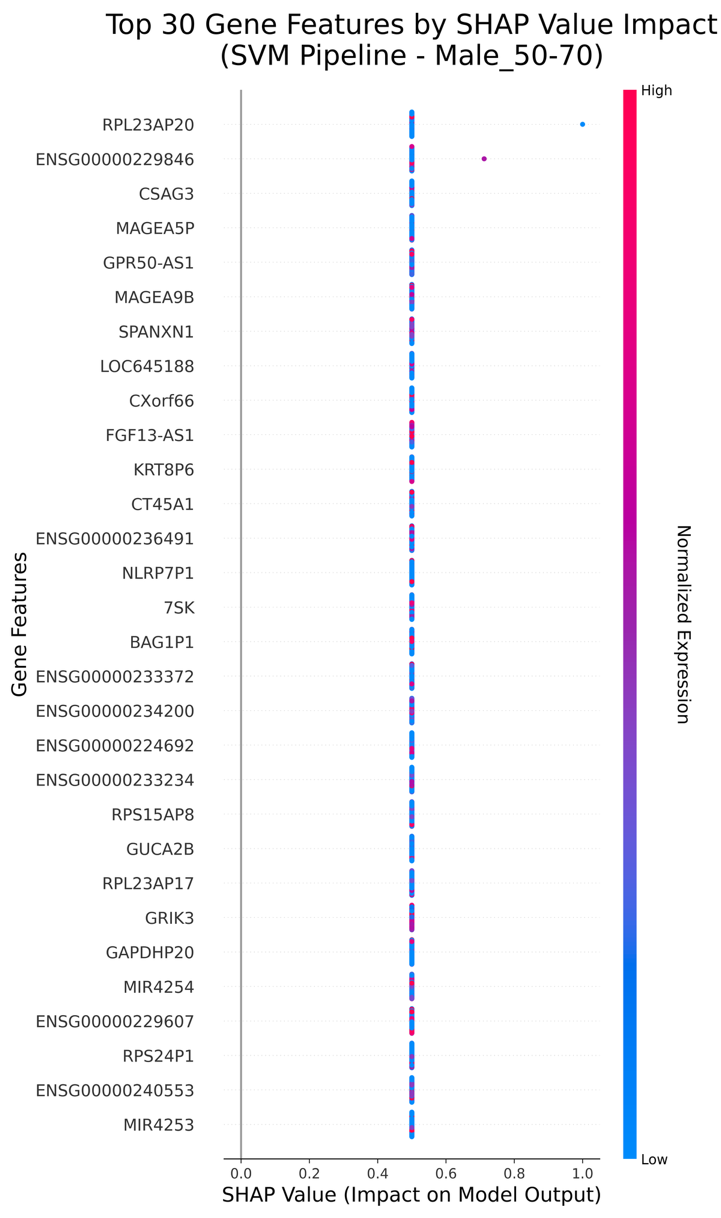

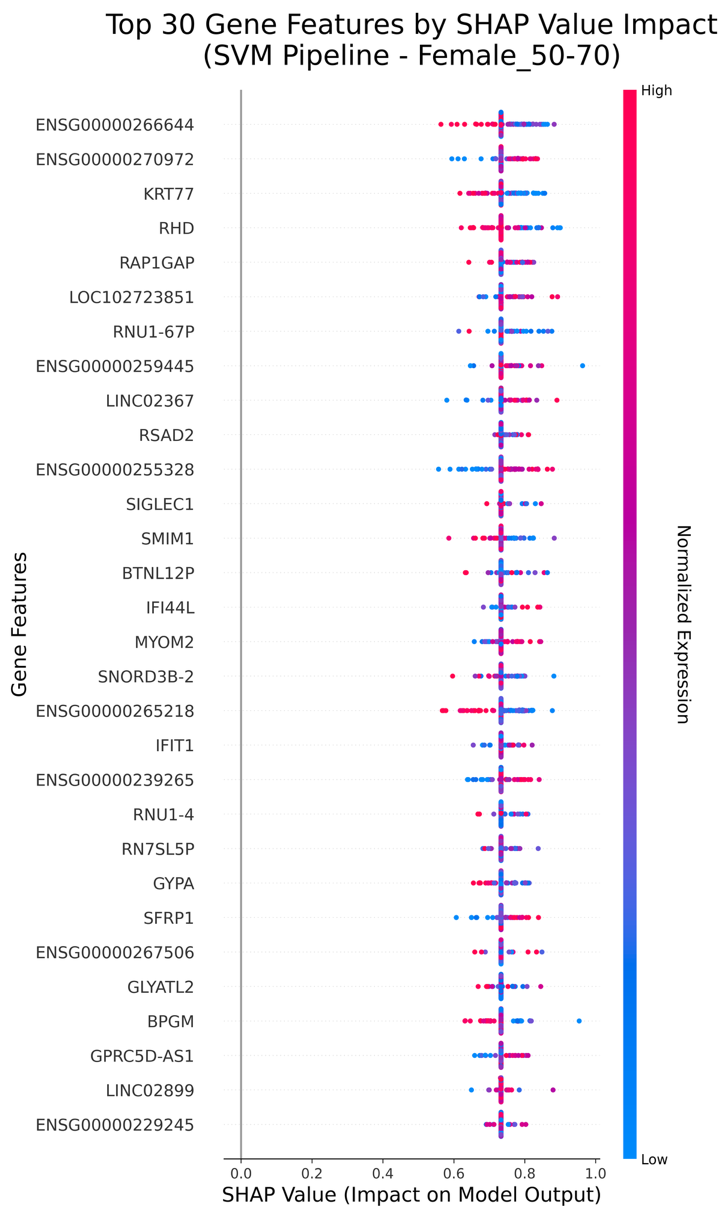


A: Males 50-70 years of age

B: Females 50-70 years of age

Figure S20: SHAP Analysis Support Vector Machine


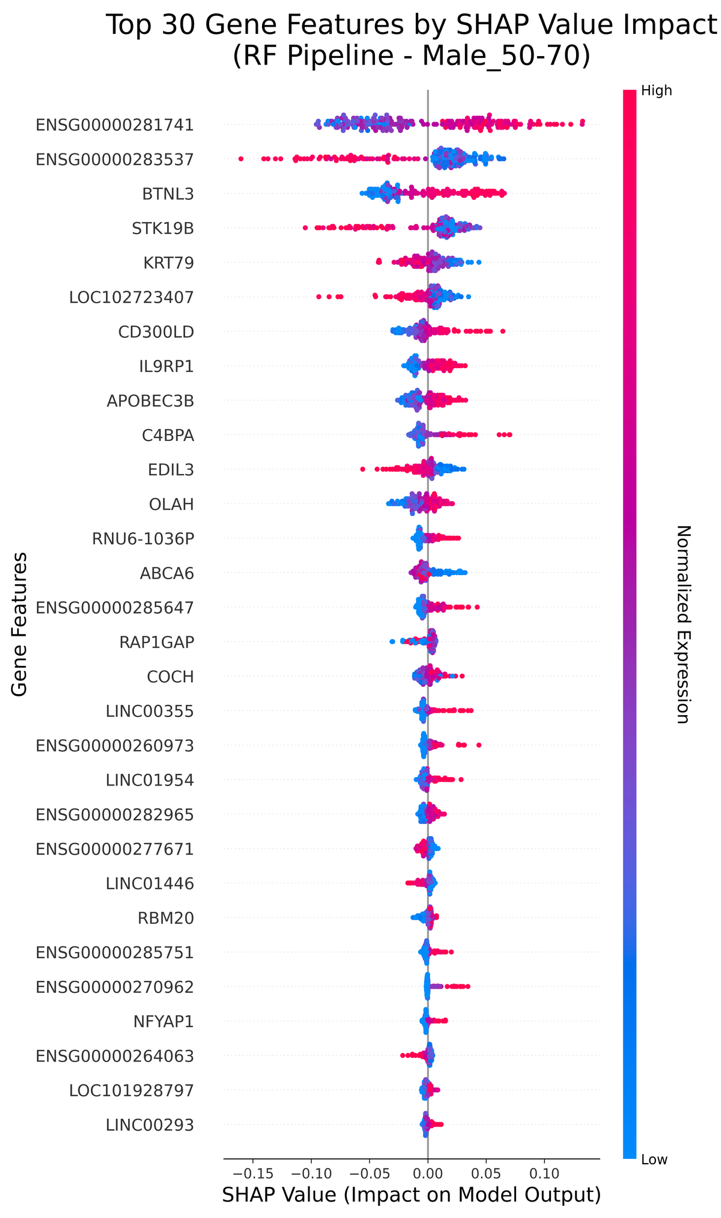

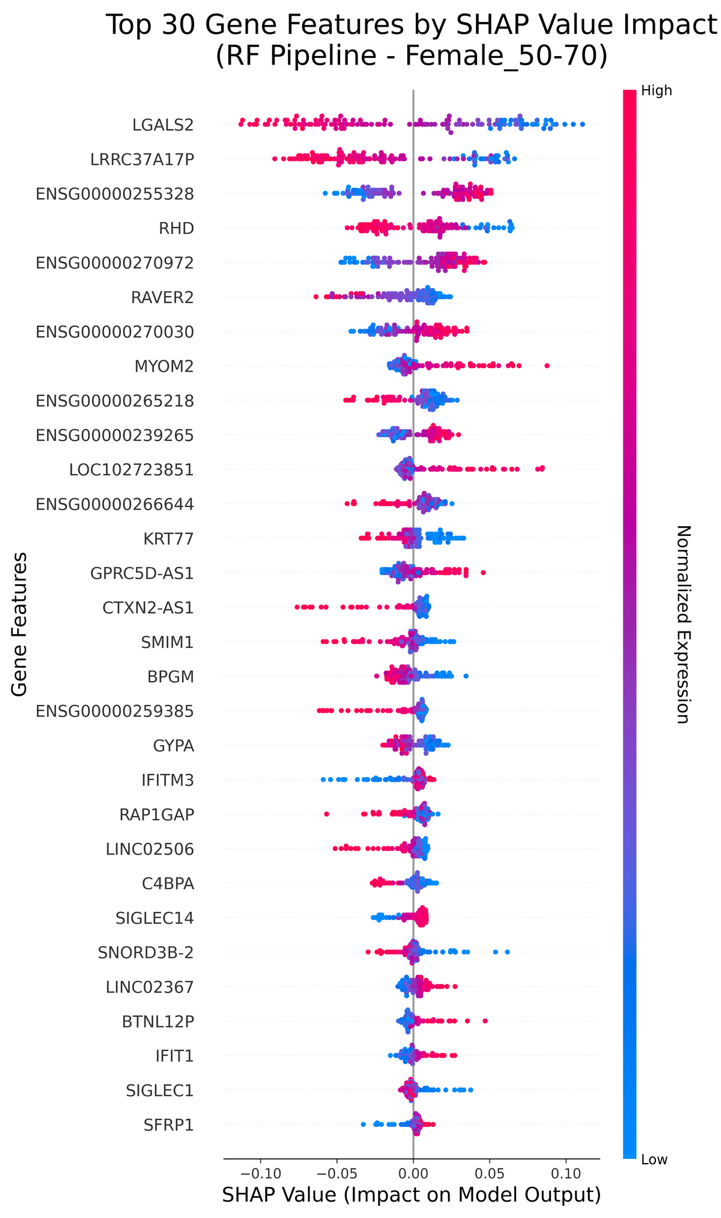


A: Males 50-70 years of age

B: Females 50-70 years of age

Figure S21: SHAP Analysis Random Forest


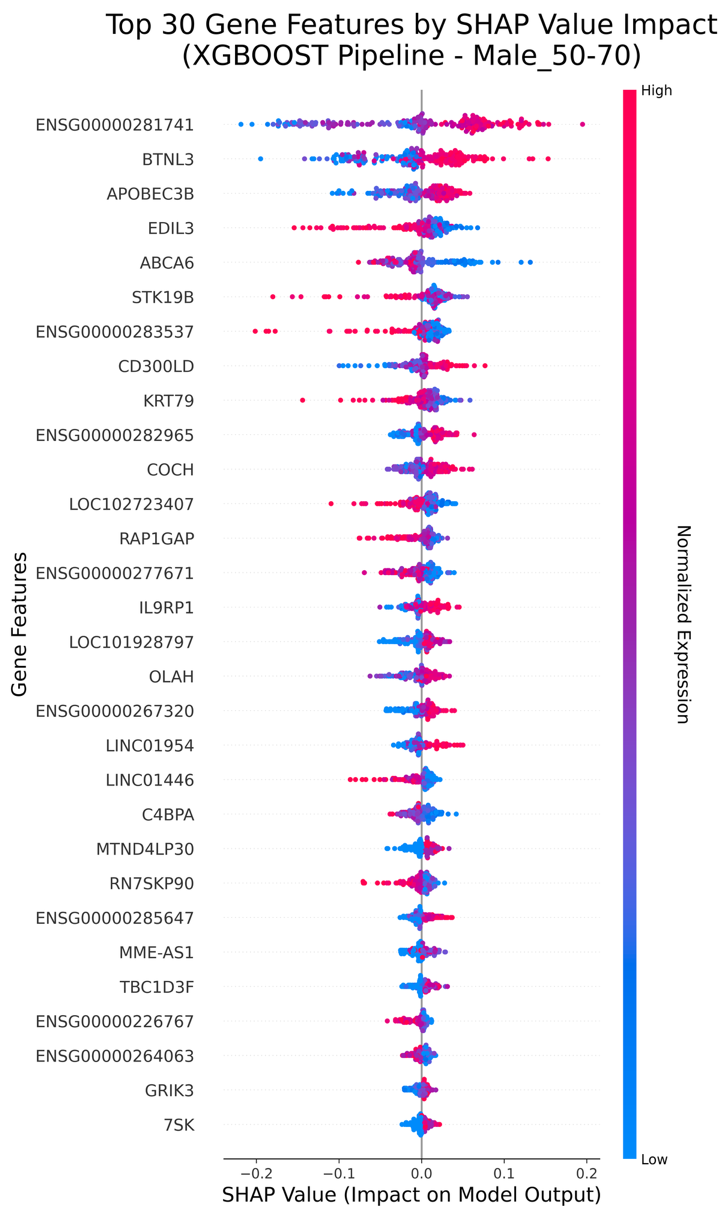

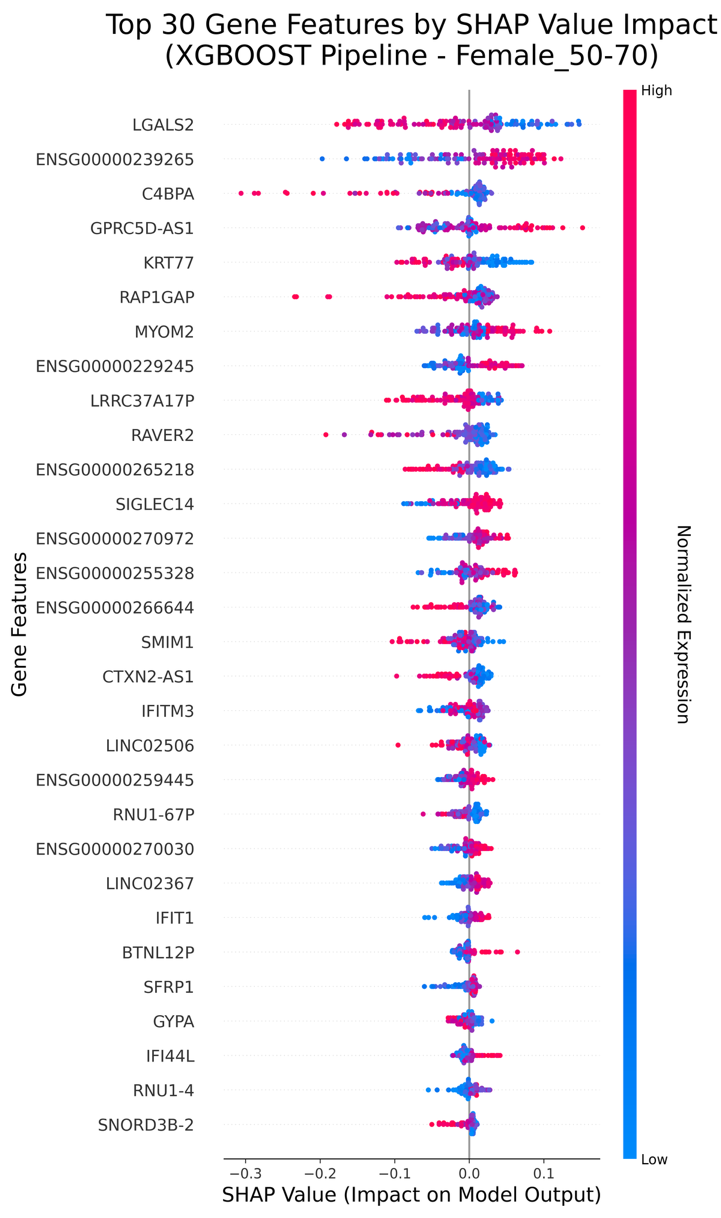


Figure S22: SHAP Analysis XGBoost

A: Males 50-70 years of age

B: Females 50-70 years of age


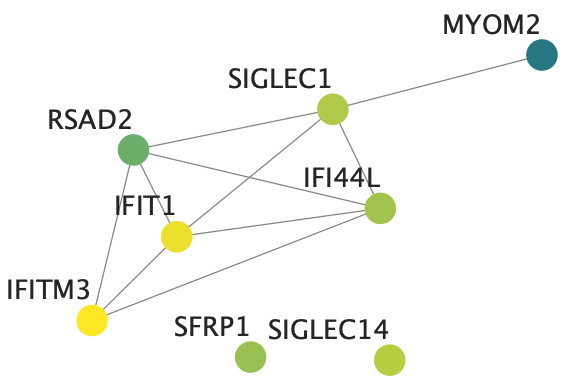


Figure S23: Females 50-70 gene network with a mostly interconnected subnetwork composed by genes linked to the immune system as constructed by Cytoscape


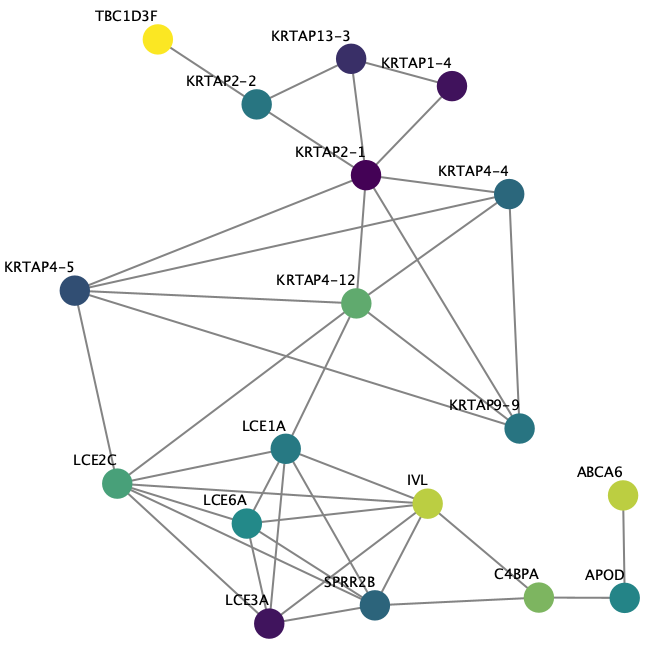


Figure S24: Males 50-70 gene network with considerable presence of keratin-like proteins as constructed by Cytoscape


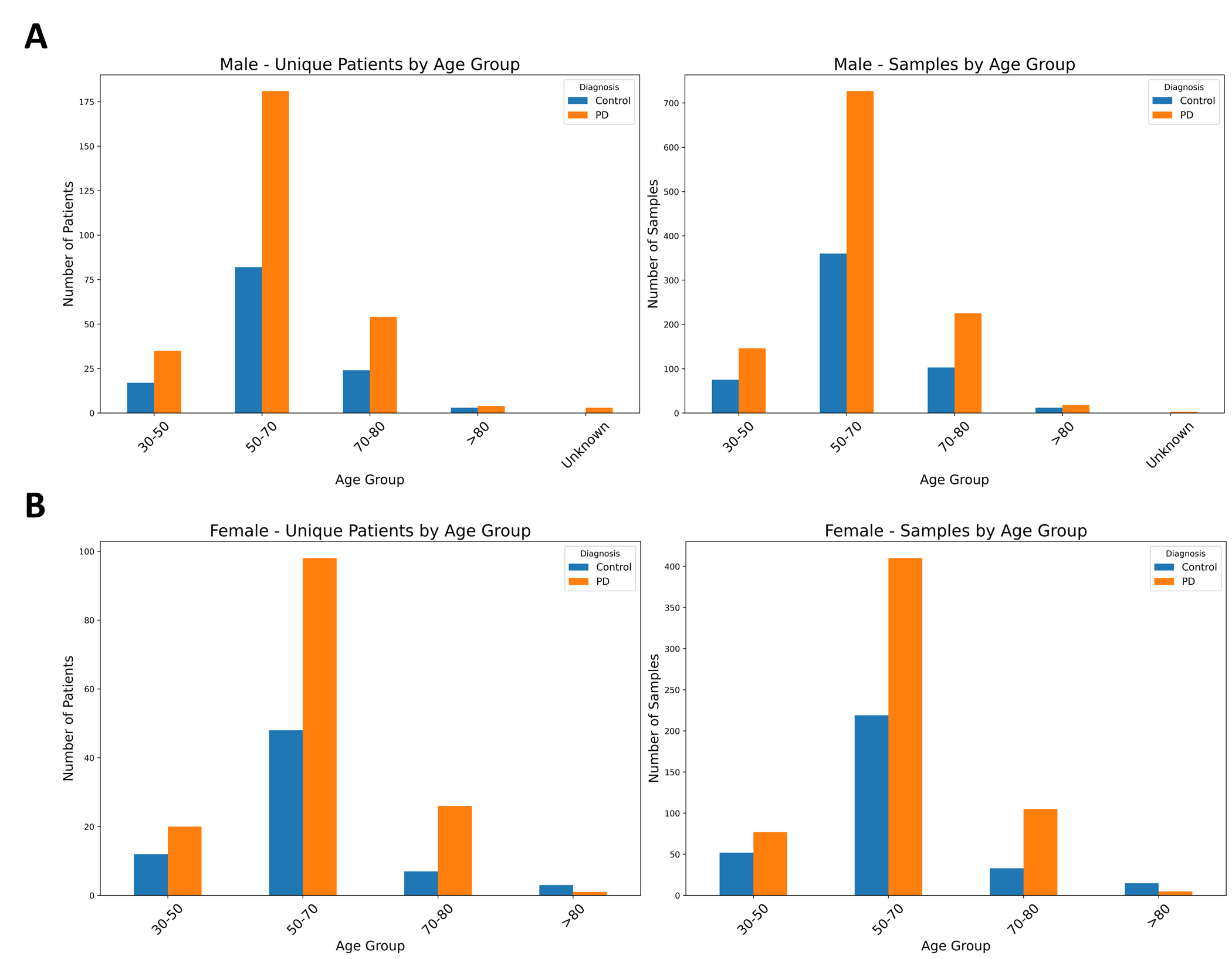


Figure S25: Distribution of Patients (left) and Samples (right) for Male (A) and Female (B) participants. The number of patients and abundance of the respective samples are depicted. Samples were gathered from the same set of participants over several visits; hence the number of actual samples is greater than the respective number of patients.

Table S1: Enrichment results for females aged 50-70 as retrieved from the STRING database. Statistically relevant results linked to immune system responses.

| **Source** | **Term Name** | **Intersecting Genes** | **P-value** |
| --- | --- | --- | --- |
| WikiPathways | Network map of SARS CoV 2 signaling | IFITM3, SIGLEC1, MYOM2 | 1*.*6 × 10^−3^ |
| Gene Ontology (BP) | biological process involved in interaction with host | IFITM3, 9606, ENSP00000371471, SIGLEC1 | 6*.*6 × 10^−3^ |
| Transfac predictions | Factor: IRF-1; motif: INRAAANNGAAASN; matc class: 1 | SIGLEC1, MYOM2 | 8*.*2 × 10^−3^ |
| mirTarBase | hsa-miR-146a-5p | IFITM3, SIGLEC1, MYOM2 | 1*.*06 × 10^−2^ |
| Gene Ontology (BP) | biological process involved in symbiotic interaction | IFITM3, l9606.ENSP00000371471, SIGLEC1 | 2*.*41 × 10^−2^ |
| Gene Ontology (BP) | defense response to virus | IFITM3, SIGLEC1, MYOM2 | 2*.*41 × 10^−2^ |
| Reactome | Interferon alpha/beta signaling | IFITM3, SIGLEC1 | 2*.*60 × 10^−2^ |
| Gene Ontology (BP) | viral life cycle | IFITM3, 9606.ENSP00000371471, SIGLEC1 | 2*.*61 × 10^−2^ |
| Transfac predictions | Factor: IRF-4; motif: KRAAANGAAAANYN; match class: 1 | SIGLEC1, MYOM2 | 3*.*91 × 10^−2^ |

Table S2: Functional Enrichment via STRING - Network for males 50-70 years of age with significant results regarding keratin across several enrichment categories

| **Category** | **Description** | **FDR value** | **Genes** |
| --- | --- | --- | --- |
| Reactome  Pathways | Keratinization | 1*.*36 × 10^−14^ | LCE1A, LC5AA, KRTAP45,  SPRR2B, IVL, LCECC,  KRTAP14, KRTAP133,  KRTAP21, KRTAP22, LC5BA |
| Pfam | Keratin, high sulfur B2 protein | 8*.*27 × 10^−11^ | KRTAP45, KRTAP14,  KRTAP44, KRTAP21,  KRTAP99, KRTAP412,  KRTAP22 |
| UniProt  Keywords | Keratinization | 1*.*06 × 10^−9^ | LCE1A, LC5AA, SPRR2B, IVL,  LCECC, LC5BA |
| STRING Clusters | Keratin | 2*.*32 × 10^−9^ | KRTAP45, KRTAP14,  KRTAP44, KRTAP133,  KRTAP21, KRTAP99,  KRTAP412 |
| GO Cellular  Component | Keratin filament | 7*.*04 × 10^−9^ | KRTAP45, KRTAP14,  KRTAP44, KRTAP21,  KRTAP99, KRTAP412,  KRTAP22 |
| GO Cellular  Component | Intermediate filament | 7*.*15 × 10^−9^ | KRTAP45, KRTAP14,  KRTAP44, KRTAP133,  KRTAP21, KRTAP99,  KRTAP412, KRTAP22 |
| STRING Clusters | Keratinization and Cornified envelope | 1*.*74 × 10^−8^ | LCE1A, LC5AA, SPRR2B, IVL,  LCECC, LC5BA |
| STRING Clusters | Keratin, high sulfur B2 protein | 1*.*95 × 10^−9^ | KRTAP45, KRTAP44,  KRTAP21, KRTAP99,  KRTAP412 |
| STRING Clusters | Keratinization | 3*.*48 × 10^−8^ | LCE1A, LC5AA, SPRR2B,  LCECC, LC5BA |
| TISSUES | Scalp | 4*.*42 × 10^−8^ | KRTAP45, KRTAP44,  KRTAP21, KRTAP99,  KRTAP412, KRTAP22 |
| STRING Clusters | Late cornified envelope | 7*.*12 × 10^−7^ | LCE1A, LC5AA, LCECC,  LC5BA |
| STRING Clusters | Keratin, high sulfur B2 protein | 1*.*01 × 10^−8^ | KRTAP45, KRTAP44,  KRTAP21, KRTAP99 |
| Reactome  Pathways | Formation of the cornified envelope | 1*.*16 × 10^−6^ | LCE1A, LC5AA, SPRR2B, IVL,  LCECC, LC5BA |
| GO Biological  Process | Keratinization | 1*.*99 × 10^−6^ | LCE1A, LC5AA, SPRR2B, IVL,  LCECC, LC5BA |
| STRING Clusters | Late cornified envelope | 9*.*07 × 10^−6^ | LCE1A, LCECC, LC5BA |
| TISSUES | Skin | 1*.*66 × 10^−5^ | KRTAP45, APOD, SPRR2B,  IVL, KRTAP44, KRTAP21,  KRTAP99, KRTAP412,  KRTAP22, LC5BA |
| UniProt  Keywords | Keratin | 6*.*13 × 10^−5^ | KRTAP45, KRTAP14,  KRTAP133, KRTAP21,  KRTAP22 |
| InterPro Domains | Keratinassociated protein | 3*.*80 × 10^−4^ | KRTAP45, KRTAP14,  KRTAP21, KRTAP22 |
| Pfam | Late cornified envelope | 9*.*70 × 10^−4^ | LCE1A, LC5AA, LCECC |
| InterPro Domains | Late cornified envelope protein | 3*.*00 × 10^−3^ | LCE1A, LC5AA, LCECC |
| STRING Clusters | Keratin, high sulfur B2 protein | 5*.*00 × 10^−3^ | KRTAP45, KRTAP44 |
| STRING Clusters | Mixed, incl. Keratin, high sulfur B2 protein, and Keratin, high-sulphur matrix protein | 5*.*00 × 10^−3^ | KRTAP21, KRTAP99 |
| GO Cellular  Component | Cytoskeleton | 2*.*38 × 10^−2^ | KRTAP45, IVL, KRTAP14,  KRTAP44, KRTAP133,  KRTAP21, KRTAP99,  KRTAP412, KRTAP22 |
| STRING Clusters | Keratinassociated protein, PMG type, and Keratinassociated protein,  type6/6/16/19/20/21 | 3*.*92 × 10^−2^ | KRTAP14, KRTAP133 |
| TISSUES | Scalp | 4*.*42 × 10^−8^ | KRTAP45, KRTAP44,  KRTAP21, KRTAP99,  KRTAP412, KRTAP22 |
| STRING Clusters | Late cornified envelope | 7*.*12 × 10^−7^ | LCE1A, LC5AA, LCECC,  LC5BA |
| STRING Clusters | Keratin, high sulfur B2 protein | 1*.*01 × 10^−8^ | KRTAP45, KRTAP44,  KRTAP21, KRTAP99 |
| Reactome  Pathways | Formation of the cornified envelope | 1*.*16 × 10^−6^ | LCE1A, LC5AA, SPRR2B, IVL,  LCECC, LC5BA |
| GO Biological  Process | Keratinization | 1*.*99 × 10^−6^ | LCE1A, LC5AA, SPRR2B, IVL,  LCECC, LC5BA |
| STRING Clusters | Late cornified envelope | 9*.*07 × 10^−6^ | LCE1A, LCECC, LC5BA |
| TISSUES | Skin | 1*.*66 × 10^−5^ | KRTAP45, APOD, SPRR2B,  IVL, KRTAP44, KRTAP21,  KRTAP99, KRTAP412,  KRTAP22, LC5BA |
| UniProt  Keywords | Keratin | 6*.*13 × 10^−5^ | KRTAP45, KRTAP14,  KRTAP133, KRTAP21,  KRTAP22 |
| InterPro Domains | Keratinassociated protein | 3*.*80 × 10^−4^ | KRTAP45, KRTAP14,  KRTAP21, KRTAP22 |
| Pfam | Late cornified envelope | 9*.*70 × 10^−4^ | LCE1A, LC5AA, LCECC |
| InterPro Domains | Late cornified envelope protein | 3*.*00 × 10^−3^ | LCE1A, LC5AA, LCECC |
| STRING Clusters | Keratin, high sulfur B2 protein | 5*.*00 × 10^−3^ | KRTAP45, KRTAP44 |
| STRING Clusters | Mixed, incl. Keratin, high sulfur B2 protein, and Keratin, high-sulphur matrix protein | 5*.*00 × 10^−3^ | KRTAP21, KRTAP99 |
| GO Cellular  Component | Cytoskeleton | 2*.*38 × 10^−2^ | KRTAP45, IVL, KRTAP14,  KRTAP44, KRTAP133,  KRTAP21, KRTAP99,  KRTAP412, KRTAP22 |
| STRING Clusters | Keratinassociated protein, PMG type, and Keratinassociated protein,  type6/6/16/19/20/21 | 3*.*92 × 10^−2^ | KRTAP14, KRTAP133 |

Table S3: Functional Enrichment via Enrichr - Indicative results for males aged 50-70 without any statistically significance according to padj yet with terms that partially overlap with pathways with a known impact in Parkinson’s disease

| **Geneset** | **Term** | **Adj. P-value** | **Genes** |
| --- | --- | --- | --- |
| SynGO_2024 | Integral Component Of Postsynaptic Density Membrane  (GO:0099061) CC | 0,9998 | GRIK3 |
| SynGO_2024 | Voltage-Gated Ca Channel Activity Involved In Regulation Of  Presynaptic Cytosolic Ca Levels (GO:0099626) BP | 0,9998 | CACNB2 |
| SynGO_2024 | Ligand-Gated IC Activity Involved In Regulation Of Presynaptic  Membrane Potential (GO:0099507) BP | 0,9998 | GRIK3 |
| SynGO_2024 | Integral Component Of Presynaptic Membrane (GO:0099056) CC | 0,9998 | GRIK3 |
| MSigDB_Hallmark_2020 | IL-2/STAT5 Signaling | 1 | COCH |
| MSigDB_Hallmark_2020 | Xenobiotic Metabolism | 1 | RAP1GAP |
| MSigDB_Hallmark_2020 | Fatty Acid Metabolism | 1 | XIST |
| MSigDB_Hallmark_2020 | Heme Metabolism | 1 | RAP1GAP |
| WikiPathways_2024_Human | Zinc Homeostasis WP3529 | 1 | MT4 |
| WikiPathways_2024_Human | Post COVID Neuroinflammation WP5485 | 1 | ACE2 |
| WikiPathways_2024_Human | SARS CoV 2 Mt Chronic Ox Stress And Endothelial Dysfunction  WP5183 | 1 | ACE2 |
| WikiPathways_2024_Human | COVID 19 Structural Coverage Map WP5145 | 1 | ACE2 |
| WikiPathways_2024_Human | RAS And Bradykinin Pathways in COVID 19 WP4969 | 1 | ACE2 |
| WikiPathways_2024_Human | Antiviral And Anti-Inflam Effects Of Nrf2 On SARS CoV 2 Pathway  WP5113 | 1 | ACE2 |
| WikiPathways_2024_Human | Type I Interferon Induction And Signaling SARS CoV 2 Infection  WP4868 | 1 | ACE2 |
| WikiPathways_2024_Human | SARS Coronavirus And Innate Immunity WP4912 | 1 | ACE2 |
| WikiPathways_2024_Human | Mitochondrial Immune Response To SARS CoV 2 WP5038 | 1 | ACE2 |
| WikiPathways_2024_Human | lncRNA In Canonical Wnt Signaling And Colorectal Cancer WP4258 | 1 | ROR1; WNT3 |
| WikiPathways_2024_Human | SARS CoV 2 Innate Immunity Evasion And Cell Immune Response  WP5039 | 1 | ACE2 |
| WikiPathways_2024_Human | Parkin Ubiquitin Proteasomal System Pathway WP2359 | 1 | TUBB8 |
| WikiPathways_2024_Human | Copper Homeostasis WP3286 | 1 | MT4 |
| WikiPathways_2024_Human | Alzheimer’s Disease WP5124 | 1 | TUBB8; WNT3 |
| WikiPathways_2024_Human | Alzheimer’s Disease And miRNA Effects WP2059 | 1 | TUBB8;  WNT3 |
| WikiPathways_2024_Human | Parkinson disease | 1 | TUBB8 |

Table S4: FDR corrected p-values from ANOVA test for bias detection.

| **Metric** | **Males** | | | | **Females** | | | | |
| --- | --- | --- | --- | --- | --- | --- | --- | --- | --- |
|  | 30-50 | 50-70 | 70-80 | >80 | 30-50 | 50-70 | 70-80 | >80 |  |
| Quantity (ug) | 7.997E-01 | 8.073E-01 | 9.024E-01 | 9.537E-01 | 8.189E-01 | 8.630E-01 | 7.396E-01 | 8.417E-01 |  |
| Concentration (ng/ul) | 7.997E-01 | 3.580E-01 | 7.723E-01 | 9.816E-01 | 9.355E-01 | 4.321E-01 | 1.705E-01 | 9.821E-01 |  |
| Concentration Flag | 7.997E-01 | 3.580E-01 | 9.016E-01 | 9.537E-01 | 9.355E-01 | 8.138E-01 | 7.930E-01 | 7.005E-01 |  |
| Submitted Volume (ul) | 7.997E-01 | 5.596E-01 | 7.723E-01 | 9.537E-01 | 9.694E-01 | 7.553E-01 | 5.768E-01 | 7.005E-01 |  |
| Normalization Volume (30ng/ul) | 7.997E-01 | 1.569E-01 | 7.723E-01 | 9.537E-01 | 8.234E-01 | 7.646E-01 | 2.089E-01 | 7.005E-01 |  |
| Total Volume (ul) | 7.997E-01 | 8.073E-01 | 9.000E-01 | 9.537E-01 | 8.189E-01 | 7.553E-01 | 7.383E-01 | 7.005E-01 |  |
| Input RNASeq (ng) | 7.997E-01 | 9.705E-01 | 9.024E-01 | 9.537E-01 | 8.234E-01 | 7.553E-01 | 8.730E-01 | 7.005E-01 |  |
| Input miRNA (ng) | 7.997E-01 | 9.705E-01 | 9.024E-01 | 9.537E-01 | 8.234E-01 | 7.553E-01 | 8.730E-01 | 7.005E-01 |  |
| 260/280 Ratio | 7.997E-01 | 3.849E-01 | 7.723E-01 | 9.816E-01 | 6.780E-01 | 8.114E-01 | 5.768E-01 | 9.126E-01 |  |
| 260/230 Ratio | 7.997E-01 | 7.564E-01 | 7.723E-01 | 9.537E-01 | 8.189E-01 | 7.807E-01 | 5.768E-01 | 9.521E-01 |  |
| deletion_length | 7.997E-01 | 6.623E-01 | 7.723E-01 | 9.816E-01 | 4.563E-01 | 1.794E-01 | 6.042E-01 | 7.005E-01 |  |
| insertion_length | 7.997E-01 | 5.596E-01 | 9.024E-01 | 9.537E-01 | 8.987E-01 | 7.646E-01 | 3.756E-01 | 6.160E-01 |  |
| mismatch_rate | 7.997E-01 | 6.623E-01 | 9.024E-01 | 9.537E-01 | 4.563E-01 | 7.807E-01 | 5.922E-01 | 7.575E-01 |  |
| multimapped_percent | 7.997E-01 | 3.849E-01 | 7.723E-01 | 9.537E-01 | 5.994E-01 | 9.430E-01 | 5.104E-01 | 6.924E-01 |  |
| uniquely_mapped_percent | 8.250E-01 | 5.596E-01 | 7.723E-01 | 9.537E-01 | 8.234E-01 | 9.430E-01 | 5.768E-01 | 7.005E-01 |  |
| num_annotated_splices | 7.997E-01 | 5.596E-01 | 7.723E-01 | 9.537E-01 | 8.189E-01 | 4.749E-01 | 2.990E-01 | 6.160E-01 |  |
| insertion_rate | 7.997E-01 | 6.623E-01 | 9.024E-01 | 9.537E-01 | 9.355E-01 | 7.553E-01 | 2.089E-01 | 9.126E-01 |  |
| unmapped_other_percent | 7.997E-01 | 9.705E-01 | 7.849E-01 | 9.537E-01 | 9.355E-01 | 7.646E-01 | 2.089E-01 | 7.005E-01 |  |
| multimapped | 8.250E-01 | 6.623E-01 | 9.024E-01 | 9.537E-01 | 8.773E-01 | 7.646E-01 | 8.833E-01 | 6.924E-01 |  |
| num_splices | 7.997E-01 | 5.596E-01 | 7.723E-01 | 9.537E-01 | 8.189E-01 | 4.749E-01 | 2.990E-01 | 6.160E-01 |  |
| avg_input_read_length | 8.715E-01 | 1.569E-01 | 7.723E-01 | 9.537E-01 | 4.563E-01 | 7.807E-01 | 1.705E-01 | 7.575E-01 |  |
| multimapped_toomany | 7.997E-01 | 9.705E-01 | 7.057E-01 | 9.537E-01 | 5.994E-01 | 9.430E-01 | 7.396E-01 | 7.005E-01 |  |
| total_reads | 7.997E-01 | 5.596E-01 | 8.622E-01 | 9.537E-01 | 5.994E-01 | 4.321E-01 | 2.089E-01 | 7.005E-01 |  |
| num_GCAG_splices | 9.881E-01 | 8.073E-01 | 7.057E-01 | 9.537E-01 | 4.563E-01 | 7.646E-01 | 8.833E-01 | 7.005E-01 |  |
| unmapped_tooshort | 7.997E-01 | 6.623E-01 | 7.723E-01 | 9.537E-01 | 4.563E-01 | 5.587E-01 | 4.333E-01 | 9.677E-01 |  |
| unmapped_other | 7.997E-01 | 8.204E-01 | 8.622E-01 | 9.537E-01 | 9.355E-01 | 9.430E-01 | 2.990E-01 | 7.005E-01 |  |
| unmapped_mismatches_percent | 7.997E-01 | 6.623E-01 | 7.723E-01 | 9.537E-01 | 8.189E-01 | 7.646E-01 | 7.396E-01 | 5.840E-01 |  |
| num_noncanonical_splices | 7.997E-01 | 9.705E-01 | 9.121E-01 | 9.537E-01 | 8.189E-01 | 7.807E-01 | 2.388E-04 | 8.724E-01 |  |
| num_ATAC_splices | 9.881E-01 | 8.204E-01 | 8.547E-01 | 9.537E-01 | 8.234E-01 | 4.749E-01 | 7.396E-01 | 6.924E-01 |  |
| multimapped_toomany_percent | 7.997E-01 | 9.705E-01 | 5.154E-01 | 9.537E-01 | 4.563E-01 | 9.430E-01 | 5.768E-01 | 7.005E-01 |  |
| avg_mapped_read_length | 7.997E-01 | 1.569E-01 | 7.723E-01 | 9.537E-01 | 5.281E-01 | 7.807E-01 | 1.705E-01 | 9.821E-01 |  |
| unmapped_mismatches | 7.997E-01 | 6.242E-01 | 7.723E-01 | 9.537E-01 | 7.083E-01 | 9.430E-01 | 5.434E-01 | 6.160E-01 |  |
| deletion_rate | 9.881E-01 | 9.705E-01 | 7.723E-01 | 9.537E-01 | 4.937E-01 | 4.321E-01 | 2.637E-01 | 5.840E-01 |  |
| uniquely_mapped | 8.250E-01 | 3.580E-01 | 7.723E-01 | 9.537E-01 | 5.994E-01 | 4.749E-01 | 2.089E-01 | 9.126E-01 |  |
| unmapped_tooshort_percent | 7.997E-01 | 9.709E-01 | 7.723E-01 | 9.816E-01 | 4.563E-01 | 9.430E-01 | 7.177E-01 | 8.724E-01 |  |
| num_GTAG_splices | 7.997E-01 | 5.596E-01 | 7.723E-01 | 9.537E-01 | 8.234E-01 | 4.749E-01 | 2.990E-01 | 6.160E-01 |  |
| PF_MISMATCH_RATE | 7.997E-01 | 5.596E-01 | 9.330E-01 | 9.816E-01 | 4.563E-01 | 7.646E-01 | 7.177E-01 | 7.487E-01 |  |
| PF_READS_ALIGNED | 7.997E-01 | 5.596E-01 | 7.723E-01 | 9.537E-01 | 7.083E-01 | 4.321E-01 | 2.089E-01 | 7.304E-01 |  |
| TOTAL_READS | 7.997E-01 | 5.596E-01 | 8.622E-01 | 9.537E-01 | 5.994E-01 | 4.321E-01 | 2.089E-01 | 7.005E-01 |  |
| PF_HQ_ERROR_RATE | 7.997E-01 | 6.623E-01 | 9.330E-01 | 9.537E-01 | 4.563E-01 | 7.807E-01 | 5.768E-01 | 7.005E-01 |  |
| PCT_CHIMERAS | 7.997E-01 | 5.726E-01 | 9.024E-01 | 9.816E-01 | 5.994E-01 | 9.430E-01 | 7.396E-01 | 7.005E-01 |  |
| PF_INDEL_RATE | 7.997E-01 | 9.705E-01 | 7.778E-01 | 9.816E-01 | 4.563E-01 | 5.144E-01 | 3.306E-01 | 7.005E-01 |  |
| PCT_PF_READS_ALIGNED | 7.997E-01 | 9.705E-01 | 7.723E-01 | 9.537E-01 | 5.994E-01 | 9.689E-01 | 9.776E-01 | 9.304E-01 |  |
| PCT_ADAPTER | 7.997E-01 | 6.623E-01 | 7.778E-01 | 9.537E-01 | 5.994E-01 | 7.882E-01 | 8.833E-01 | 7.005E-01 |  |
| MEAN_INSERT_SIZE | 7.997E-01 | 7.564E-01 | 7.723E-01 | 9.537E-01 | 8.189E-01 | 8.223E-01 | 2.089E-01 | 9.409E-01 |  |
| PCT_UTR_BASES | 7.997E-01 | 3.849E-01 | 7.723E-01 | 9.816E-01 | 7.188E-01 | 7.646E-01 | 2.990E-01 | 5.840E-01 |  |
| PCT_INTERGENIC_BASES | 7.997E-01 | 6.623E-01 | 7.057E-01 | 9.537E-01 | 7.651E-01 | 7.646E-01 | 9.564E-01 | 7.005E-01 |  |
| MEDIAN_CV_COVERAGE | 7.997E-01 | 9.705E-01 | 2.891E-01 | 9.816E-01 | 4.563E-01 | 7.646E-01 | 1.705E-01 | 7.005E-01 |  |
| PCT_CODING_BASES | 8.250E-01 | 3.339E-01 | 9.024E-01 | 9.537E-01 | 5.994E-01 | 7.807E-01 | 8.115E-01 | 7.005E-01 |  |
| MEDIAN_3PRIME_BIAS | 7.997E-01 | 9.705E-01 | 7.275E-01 | 9.816E-01 | 7.188E-01 | 7.553E-01 | 5.768E-01 | 7.005E-01 |  |
| NUM_UNEXPLAINED_READS | NA | 9.621E-01 | 7.723E-01 | 9.816E-01 | 8.358E-01 | 4.749E-01 | 5.768E-01 | 7.005E-01 |  |
| PCT_RIBOSOMAL_BASES | 9.881E-01 | 7.564E-01 | NA | NA | NA | NA | NA | NA |  |
| PCT_INTRONIC_BASES | 7.997E-01 | 3.339E-01 | 7.723E-01 | 9.537E-01 | 9.355E-01 | 7.807E-01 | 2.089E-01 | 6.160E-01 |  |
| PCT_R2_TRANSCRIPT_STRAND_READS | 8.250E-01 | 8.073E-01 | 7.275E-01 | 9.537E-01 | 5.994E-01 | 7.807E-01 | 9.824E-01 | 5.840E-01 |  |
| PCT_MRNA_BASES | 7.997E-01 | 3.339E-01 | 7.723E-01 | 9.816E-01 | 8.234E-01 | 7.553E-01 | 3.197E-01 | 5.840E-01 |  |
| MEDIAN_5PRIME_TO_3PRIME_BIAS | 7.997E-01 | 9.705E-01 | 7.723E-01 | 9.537E-01 | 4.563E-01 | 5.587E-01 | 7.383E-01 | 9.821E-01 |  |
| PCT_R1_TRANSCRIPT_STRAND_READS | 7.997E-01 | 8.073E-01 | 7.275E-01 | 9.537E-01 | 5.994E-01 | 7.807E-01 | 9.824E-01 | 5.840E-01 |  |
| MEDIAN_5PRIME_BIAS | 7.997E-01 | 7.564E-01 | 8.622E-01 | 9.537E-01 | 4.563E-01 | 7.646E-01 | 8.833E-01 | 7.005E-01 |  |
| PCT_USABLE_BASES | 7.997E-01 | 3.422E-01 | 7.723E-01 | 9.816E-01 | 9.078E-01 | 7.553E-01 | 3.306E-01 | 5.840E-01 |  |
| RIN Value | 7.997E-01 | 5.726E-01 | 7.723E-01 | 9.537E-01 | 4.563E-01 | 7.807E-01 | 7.177E-01 | 9.304E-01 |  |

Table S5: Locations of Source Code. The relevant scripts are submitted in GitHub as well as made permanently available for downloads on Zenodo and accessible via Digital Object Identifier.

| Location | URL |
| --- | --- |
| Source Code Repository | https://github.com/perpk/comp-analysis-rna-data-netw-pd |
| Permanent Link to Source Code on Zenodo | https://doi.org/10.5281/zenodo.17290094 |
